# Identification of microRNAs and Their Target Genes Explores miRNA-Mediated Regulatory Network of Cytoplasmic Male Sterility Occurrence during Anther Development in Radish (*Raphanus sativus* L.)

**DOI:** 10.3389/fpls.2016.01054

**Published:** 2016-07-22

**Authors:** Wei Zhang, Yang Xie, Liang Xu, Yan Wang, Xianwen Zhu, Ronghua Wang, Yang Zhang, Everlyne M. Muleke, Liwang Liu

**Affiliations:** ^1^National Key Laboratory of Crop Genetics and Germplasm Enhancement, College of Horticulture, Nanjing Agricultural UniversityNanjing, China; ^2^Department of Plant Sciences, North Dakota State UniversityFargo, ND, USA

**Keywords:** radish (*Raphanus sativus* L.), cytoplasmic male sterility, microRNA, target gene, qRT-PCR, high-throughput sequencing

## Abstract

MicroRNAs (miRNAs) are a type of endogenous non-coding small RNAs that play critical roles in plant growth and developmental processes. Cytoplasmic male sterility (CMS) is typically a maternally inherited trait and widely used in plant heterosis utilization. However, the miRNA-mediated regulatory network of CMS occurrence during anther development remains largely unknown in radish. In this study, a comparative small RNAome sequencing was conducted in floral buds of CMS line ‘WA’ and its maintainer line ‘WB’ by high-throughput sequencing. A total of 162 known miRNAs belonging to 25 conserved and 24 non-conserved miRNA families were isolated and 27 potential novel miRNA families were identified for the first time in floral buds of radish. Of these miRNAs, 28 known and 14 potential novel miRNAs were differentially expressed during anther development. Several target genes for CMS occurrence-related miRNAs encode important transcription factors and functional proteins, which might be involved in multiple biological processes including auxin signaling pathways, signal transduction, miRNA target silencing, floral organ development, and organellar gene expression. Moreover, the expression patterns of several CMS occurrence-related miRNAs and their targets during three stages of anther development were validated by qRT-PCR. In addition, a potential miRNA-mediated regulatory network of CMS occurrence during anther development was firstly proposed in radish. These findings could contribute new insights into complex miRNA-mediated genetic regulatory network of CMS occurrence and advance our understanding of the roles of miRNAs during CMS occurrence and microspore formation in radish and other crops.

## Introduction

MicroRNAs (miRNAs) are a type of endogenous non-coding small RNAs of ~21–24 nucleotides that are known to be important negative regulators of gene expression at transcriptional and post-transcriptional level by mediating mRNA degradation or translational repression (Voinnet, 2009). In plants, primary miRNAs (pri-miRNAs) are transcribed from nuclear-encoded *MIR* genes by RNA polymerase II and cleaved by Dicer-like1 (DCL1) assisted by the dsRNA binding protein HYL1 to generate miRNA:miRNA^*^ duplexes called pre-miRNAs (Jones-Rhoades et al., 2006; Kurihara et al., 2006; Ruiz-Ferrer and Voinnet, 2009). The duplexes are then methylated by HEN1 and one of the strands combines with the argonaute protein1 (AGO1) to form the RNA-induced silencing complex (RISC), which regulates gene expression through mRNA degradation with nearly perfect complementarity or translational repression with partial complementarity (Yu et al., 2005; Jones-Rhoades et al., 2006; Bodersen et al., 2008).

Cytoplasmic male sterility (CMS) is a maternally inherited trait in plant, which is unable to produce functional pollen, and is a widely observed phenomenon in nearly 200 species (Brown et al., 2003; Hu et al., 2012). CMS lines have been widely used for the production of F_1_ hybrid seeds and utilization of heterosis in many crops, such as cotton, maize, sorghum, wheat, rice, beet, and rapeseed (Schnable and Wise, 1998; Bentolila et al., 2002; Kubo et al., 2011). In addition to its crucial breeding tools, CMS lines also provide important materials for studying anther and pollen development, and cytoplasmic-nuclear interactions (Chen and Liu, 2014). CMS is usually due to the effect of sterilizing factors found in the mitochondrial genome (Touzet and Meyer, 2014). In most cases, CMS can be restored by nuclear-encoded fertility restorer (*Rf*) gene(s), which relies on *Rf* suppressing cytoplasmic dysfunction caused by mitochondrial genes (Eckardt, 2006). High-throughput sequencing now is widely used and has been proven to be an excellent application for the identification of plant miRNAs. As a class of negative regulators, miRNAs have also been identified and characterized during anther development in several plant species, including *Arabidopsis* (Chambers and Shuai, 2009), *Oryza sativa* (Wei et al., 2011; Yan et al., 2015), *Gossypium hirsutum* (Wei et al., 2013), *Brassica juncea* (Yang et al., 2013), and *B. rapa* (Jiang et al., 2014). In *G. hirsutum*, 16 conserved miRNA families were identified during anther development between the Genetic male sterility (GMS) mutant and its wild type. In *O. sativa*, Wei et al. (2011) identified 292 known miRNAs and 75 novel miRNAs from sporophytic tissues and pollen at three developmental stages. Additionally, many CMS occurrence-associated miRNAs have also been identified in some vegetable crops. In *B. rapa*, 54 conserved and eight novel miRNA families involved in pollen development were identified (Jiang et al., 2014). In *B. juncea*, 197 known and 93 new candidate miRNAs during pollen development between CMS line and its maintainer line were also identified (Yang et al., 2013). Although a large number of miRNAs during anther development have been isolated and identified in many crop species, the miRNA-mediated regulatory network of CMS occurrence during anther development remain to be clarified in root vegetable crops.

Radish (*Raphanus sativus* L. 2*n* = 2*x* = 18) is an important annual or biennial root vegetable crop of Brassicaceae family. In recent years, some conserved and novel miRNAs associated with taproot thickening, embryogenesis, flowering-time, and heavy metal stresses had been widely identified in radish (Xu et al., 2013a; Zhai et al., 2014; Nie et al., 2015; Wang et al., 2015; Yu et al., 2015). However, there is little information about the CMS occurrence at the post-transcriptional level in radish. To systematically explore the roles of miRNAs and their targets involved in CMS occurrence during anther development in radish, two small RNA libraries from ‘WA’ (male sterile line), and ‘WB’ (maintainer, fertile line) floral buds of radish were constructed. The aims of this study were to identify known and potential novel miRNAs from the two libraries and investigate the dynamic expression patterns of the CMS occurrence-related miRNAs and their targets during anther development in radish plant. Furthermore, the miRNA-mediated regulatory network of CMS occurrence during anther development was constructed in radish. These results would lay a valuable foundation for elucidating the regulatory roles of CMS occurrence-related miRNAs in radish and facilitate further dissection of the molecular mechanisms underlying microspore formation and CMS occurrence in other crops.

## Materials and methods

### Plant materials

The radish cytoplasmic male sterile line ‘WA’ and its maintainer line ‘WB’ were used as materials in this study. The ‘WB’ was advanced inbred line through multiple self-pollination for more than 10 generations, while CMS line ‘WA’ was developed through continuously backcrossing with ‘WB’ for more than 10 generations. ‘WA’ had completely aborted anthers without pollen, whereas ‘WB’ had normal anthers with fertile pollen (Figure S1). The materials were planted under normal conditions at Jiangpu Breeding Station of Nanjing Agricultural University, China. According to the cytological characterization of the developmental stages identified with paraffin section technique, the longitudinal length of floral buds reaching 1–1.5, 2–2.5, and 4–5 mm corresponds to the stage of meiosis, tetrad, and early microscope, respectively (Figure S1), which was in highly accordance with results of previous studies (Sun et al., 2012, 2013). Floral buds at three stages were independently collected from the two lines with three biological replicates. Each sample was collected from three randomly selected individual plants and immediately frozen in liquid nitrogen and stored at −80°C for further use.

### High-throughput sequencing of small RNAs

Total RNAs were extracted from three stages of floral buds of ‘WA’ and ‘WB’ using Trizol® Reagent (Invitrogen, USA) according to the manufacturer's protocols, respectively. RNAs from the three different stages were equally pooled and used for two small RNA libraries (WA and WB) construction according to previously described procedures (Hafner et al., 2008; Xu et al., 2013b). In brief, small RNA fractions of 18–30 nt were separated and purified from total RNA by 15% denaturing polyacrylamide gel electrophoresis. Then the isolated sRNAs were ligated to 5′ and 3′ adaptors and reverse transcribed to cDNA through SuperScript II Reverse Transcriptase (Invitrogen) and amplified by PCR. Finally, sRNA libraries were sequenced by the Solexa sequencer (Illumina) HiSeq^TM^ 2500.

The clean reads were obtained after removing low quality reads, reads with 5′ primer contaminants or poly-A tails, trimming reads smaller than 18 nt or longer than 30 nt. The remaining unique sequences were then mapped to the radish reference sequences including genomic survey sequences (GSS), expressed sequence tag (EST) sequences and the radish mRNA transcriptome sequences (accession number: SRX1671013) using the SOAP2 program (Li et al., 2009; Xu et al., 2013a). Only perfect matched sequences with no more than two mismatches were retained for proceeding analysis. After using BLAST in GenBank (http://www.ncbi.nlm.nih.gov/genbank/) and Rfam 12.0 (http://rfam.xfam.org/) database, the clean reads compared with the non-coding RNAs (rRNAs, tRNAs, snRNAs, and snoRNA) were removed for further analysis. The remaining matched reads were aligned with known miRNAs in miRBase 21 (http://www.mirbase.org/index.shtml) for radish known miRNAs identification. Then, the unannotated reads were used to predict potential novel miRNAs using Mireap software (https://sourceforge.net/projects/mireap/) according to the previous criteria (Meyers et al., 2008). The stem-loop structure of miRNA precursors were folded by Mfold (http://unafold.rna.albany.edu/?q=mfold/RNA-Folding-Form) (Zuker, 2003).

### Differential expression analysis of miRNAs between CMS line and its maintainer line

The frequency of miRNAs from two libraries was normalized to one million by total number of miRNAs per sample (Gao et al., 2012). If the normalized read of a given miRNA is zero, the expression value was set to 0.01 for further use. The differential expression of miRNAs between the two libraries was calculated as: Fold-change = log_2_ (WA/WB). The *P*-value was calculated following the previously reported methods (Li et al., 2009; Zhai et al., 2014). The miRNAs with *P* ≤ 0.05 and fold-change ≥ 1 or ≤ −1 were considered as up- or down-regulated miRNAs between the two libraries during anther development, respectively.

### Prediction and annotation of potential targets for CMS occurrence-related miRNAs

The potential target genes of the identified miRNAs were predicted by the plant small RNA target analysis server (psRNATarget; http://plantgrn.noble.org/psRNATarget/) (Dai and Zhao, 2011). The criteria used for target prediction in plants were performed following previous methods (Allen et al., 2005). To understand the biological functions of the targets, gene ontology (GO) analysis were performed by Blast2GO program on the basis of the BLAST searching against the available Nr database in NCBI. In addition, KEGG Orthology Based Annotation System (KOBAS2.0; http://kobas.cbi.pku.edu.cn/home.do) was applied to predict the biological functions of target genes (Xie et al., 2011). Based on the differentially expressed miRNAs and their corresponding targets, the miRNA-targets regulatory network was constructed using Cytoscape_v3.2.1 software (Smoot et al., 2011).

### qRT–PCR validation

Quantitative reverse transcription-PCR (qRT–PCR) was employed to evaluate the validity of small RNA sequencing and also to analyze the expression patterns of miRNAs and their targets during different stages. miRNAs and total RNAs were extracted from samples and reverse-transcribed to cDNA using the One Step Primer Script® miRNA cDNA Synthesis Kit (Takara Bio Inc., Dalian, China) and SuperScript® III Reverse Transcriptase (Invitrogen, USA) following the manufacturer's instructions, respectively. All reactions were performed on a BioRad iQ5 sequence detection system (BIO-RAD) and carried out in a total volume of 20 μl including 0.2 μM primer pairs, 2 μl diluted cDNA, and 10 μl 2 × SYBR Green PCR Master Mix (TaKaRa). The PCR amplification reaction was performed following the previous reports (Zhai et al., 2014). The 5.8S ribosomal RNA (rRNA) was used as the reference gene for normalization. All reactions were done in triplicate, the 2^−ΔΔ*C*_T_^ method was used to calculate the relative expression data (Livak and Schmittgen, 2001). The statistical analysis was performed using SPSS 20 software (SPSS Inc., USA) with Duncan's multiple range test at the 5% level of significance. The primers for qRT–PCR were showed in Table S1.

## Results

### Overview analysis of sequences from small RNA libraries

To identify known and potential novel miRNAs involved in anther development and CMS occurrence, we constructed two small RNA libraries from the floral buds of ‘WA’ and ‘WB’ line. A total of 43,068,458 raw reads were obtained from the two sRNA libraries. After filtering low quality reads, adapter contaminants, and reads smaller than 18 nucleotides, we obtained 20,287,225 (representing 5,528,061 unique sequences), and 21,989,236 (representing 5,682,107 unique sequences) clean reads from WA and WB library, respectively (Table S2). Of these reads, 13.84% were WA library-specific with 42.68% unique sRNAs, 13.88% were WB library-specific with 44.24% unique sRNAs, and 72.28% were present in both with 13.08% unique sRNAs (Table S3).

**Figure 1:**
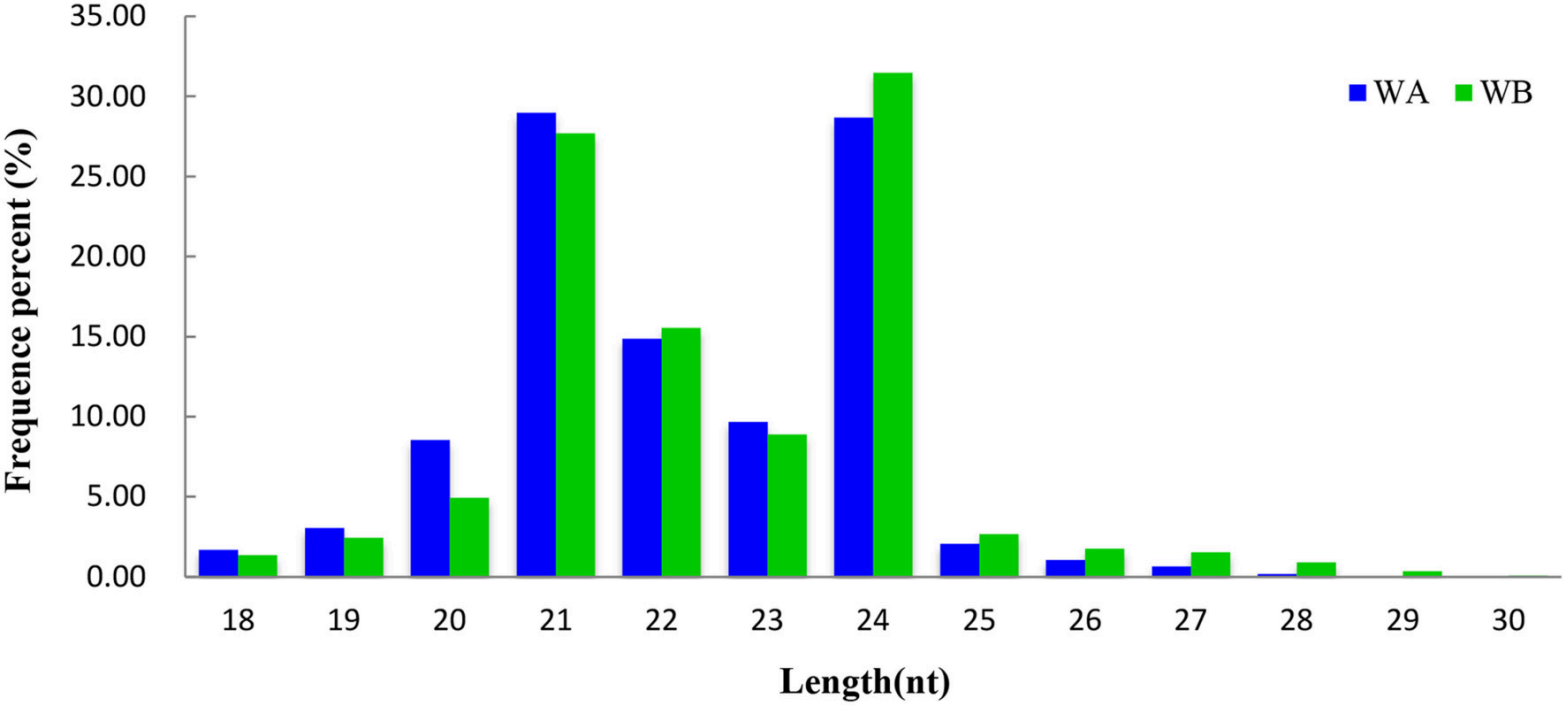
**Length distribution of small RNAs in WA and WB library**.

By comparing with the NCBI GenBank and Rfam databases, these clean reads that matched non-coding sRNAs including rRNAs, snoRNAs, snRNAs, and tRNAs were eliminated. After that, 27,092 (WA) and 27,764 (WB) unique sequences were acquired by querying the unique reads against miRBase 21 (Table 1). The remaining 5,388,388 (WA) and 5,511,728 (WB) unannotated unique reads were used for identification of potential novel miRNAs (Table 1). The length distribution of sRNA reads ranged from 18 to 30 nt in both libraries (Figure 1), and the most abundant sequences in the two libraries ranged from 20 to 24 nt, which is the representative size range of products cleaved by DCLs (Henderson et al., 2006). The most abundant sRNAs in WA and WB library was 21 and 24 nt long, which accounted for 28.97 and 31.47%, respectively.

**Table 1:** **Distribution of small RNAs among different categories in radish**.

**Category**	**WA**		**WB**	
	**Unique sRNAs**	**Total sRNAs**	**Unique sRNAs**	**Total sRNAs**
Total	5528061 (100%)	20287225 (100%)	5682107 (100%)	21989236 (100%)
miRNA	27092 (0.49%)	1453994 (7.17%)	27764 (0.49%)	1521955 (6.92%)
rRNA	92089 (1.67%)	2685339 (13.24%)	117885 (2.07%)	3446274 (15.67%)
snRNA	6099 (0.11%)	22072 (0.11%)	6602 (0.12%)	27969 (0.13%)
snoRNA	3383 (0.06%)	10011 (0.05%)	4120 (0.07%)	11837 (0.05%)
tRNA	11010 (0.20%)	1157890 (5.71%)	14008 (0.25%)	514526 (2.34%)
unannotated	5388388 (97.47%)	14957919 (73.73%)	5511728 (97%)	16466675 (74.89%)

### Identification of known miRNAs during anther development

To identify known miRNAs from the two libraries, the unique sRNA reads were aligned to known miRNA precursors, and mature miRNA sequences in miRBase 21, allowing a maximum of two mismatches. A total of 124 unique reads belonging to 25 conserved miRNA families were identified in the two libraries (Table 2). The distribution of conserved miRNA family members was analyzed (Figure S2). A large part of conserved miRNA families had members of more than three, and miR165/166 family possessed the largest member of 17, followed by miR156/157, and miR169 with 14 and 11 members, respectively. However, some conserved miRNA families including miR158, miR161, miR391, miR395, miR397 miR398, and miR403 had only one or two members. In addition, 38 unique reads belonging to 24 non-conserved miRNA families were also discovered in these two libraries, which contained fewer members as compared with conserved miRNAs (Figure S2).

**Table 2:** **Known miRNA families and their expression abundance in WA and WB library**.

**Family**	**Number of members**	**miRNA reads**		**Total reads**	**Ratio (WA/WB)**
		**WA**	**WB**		
**CONSERVED miRNA**					
miR156/157	14	410237	304695	714932	1.35
miR158	2	15565	7641	23206	2.04
miR159	4	1726	1157	2883	1.49
miR160	6	13463	18893	32356	0.71
miR161	1	152	0	152	–
miR162	3	1211	1379	2590	0.88
miR164	5	9831	9100	18931	1.08
miR165/166	17	321161	405255	726416	0.79
miR167	8	318811	276963	595774	1.15
miR168	3	137677	135624	273301	1.02
miR169	11	36871	37179	74050	0.99
miR171	6	3241	4824	8065	0.67
miR172	7	9866	13246	23112	0.74
miR319	4	508	976	1484	0.52
miR390	5	7594	10560	18154	0.72
miR391	2	3769	3177	6946	1.19
miR393	3	1789	1643	3432	1.09
miR394	3	358	348	706	1.03
miR395	2	45236	44	45280	1028.09
miR396	6	3751	2853	6604	1.31
miR397	1	13	60	73	0.22
miR398	2	223	285	508	0.78
miR399	3	108	80	188	1.35
miR403	2	1455	1306	2761	1.11
miR408	4	3431	7780	11211	0.44
**NON-CONSERVED miRNA**					
miR400	2	43	33	76	1.30
miR447	1	189	245	434	0.77
miR482	2	2073	35	2108	59.23
miR529	3	68	55	123	1.24
miR535	2	2229	83	2312	26.86
miR824	2	651	681	1332	0.96
miR825	1	354	479	833	0.74
miR827	3	1932	2558	4490	0.76
miR828	1	5	71	76	0.07
miR829	1	36	98	134	0.37
miR831	1	87	58	145	1.50
miR845	3	5094	5262	10356	0.97
miR854	1	640	468	1108	1.37
miR858	1	0	26	26	0.00
miR859	1	671	0	671	–
miR860	1	248	215	463	1.15
miR948	1	0	336	336	0.00
miR1878	1	0	1584	1584	0.00
miR1885	2	1961	4902	6863	0.40
miR2111	4	91	206	297	0.44
miR2118	1	42998	60406	103404	0.71
miR2199	1	42085	44471	86556	0.95
miR3444	1	0	452	452	0.00
miR5654	1	1298	1705	3003	0.76

The number of miRNA reads differed greatly in the two libraries (Figure S3). For instance, miR156/157 presented the highest expression abundance with 410,237 in WA library, while miRNA165/166 displayed the highest expression of 405,255 copies in WB library. Several miRNA families such as miR167, miR168, miR2118, and miR2199 also displayed extraordinarily high abundance in both libraries, while some other miRNA families (miR400, miR828, miR829, miR831, and miR858) were expressed with relatively low levels of expression with no more than 100 reads in WA and WB library. In addition, the expression levels of different members of the same miRNA family varied drastically (Table S4).

### Identification of potential novel miRNAs in floral buds

A total of 30 precursor sequences and 27 novel miRNA families were identified in the two libraries (Table S5). The secondary structures of these predicted novel miRNA precursors were displayed in Figure S4. In addition to secondary structure prediction, identification of complementary sequences of the mature miRNAs is another way to provide forceful evidences for these predicted novel miRNAs (Meyers et al., 2008). Out of these potential novel miRNAs, only seven miRNAs with mature and complementary miRNA^*^s were detected as the novel miRNA candidates (Table 3). In the present study, the length of these mature miRNAs ranged from 20 to 23 nt, with a distribution peak at 21 nt (60.0%). Furthermore, the length of these potential novel miRNA precursors ranged from 72 to 255 nt with the average length of 142.6 nt. The minimum free energy (MFE) value ranged from −97.83 to −22.9 kcal/mol with an average value of −48.32 kcal/mol. In addition, nine potential novel miRNAs were expressed in both libraries, while a total of 14 and 8 potential novel miRNAs were WA library-specific, and WB library-specific, respectively (Table S5). Most of these potential novel miRNAs had relatively low expression levels when compared with known miRNAs, and the expression levels of miRNA^*^ sequences were obviously less than those of their corresponding mature miRNAs, which was consistent with the viewpoint that miRNA^*^ strands degraded quickly during the biogenesis of mature miRNAs (Rajagopalan et al., 2006).

**Table 3:** Novel miRNAs with their complementary miRNA^*^s during anther development in radish.

**miRNA name**	**Reads**		**Mature sequence (5′–3′)**	**Arm**	**Size**	**LP (nt)**	**MFE (kcal/mol)**	**miRNA location**
	**WA**	**WB**						
rsa-miRn3	443	0	TATTCCGACGACAATTCCGACG	5′	22	100	–56.31	CL2831.Contig4,Contig6
rsa-miRn3^*^	8	0	TCGGAATTCCGTCGGAATATA	3′	21	100	–56.31	CL2831.Contig4,Contig6
rsa-miRn4	519	637	AATGTATGTAGTCCAATCTAT	5′	21	117	–66	CL2870.Contig2
rsa-miRn4^*^	18	6	ACATTGGACTACATATATTAC	3′	21	117	–66	CL2870.Contig2
rsa-miRn5	1141	1452	GCTTCCATATCTAGCAGTAGG	5′	21	184	–75.8	CL2916.Contig2
rsa-miRn5^*^	6	12	TACCGATAGATGTGGAAGCGT	3′	21	184	–75.8	CL2916.Contig2
rsa-miRn7	21141	24827	TTTGCGTGAGTATGTGGATGT	5′	21	119	–49	CL4600.Contig2
rsa-miRn7^*^	32	42	ATCCACATACTCACGAAAATC	3′	21	119	–49	CL4600.Contig2
rsa-miRn9a	771	1062	CGTTCAGTTCTCCTTTTGCTTC	5′	22	106	–47.24	Rsa#S43006900
rsa-miRn9a^*^	7	28	AGCAAACGAGAATTGAACGGA	3′	21	106	–47.24	Rsa#S43006900
rsa-miRn19	0	186	GAACGATATAAAAGATCATGGA	5′	22	105	–30.2	CL6156.Contig1, Contig2
rsa-miRn19^*^	0	44	TATGGCCTTTATATCGTATTCG	3′	22	105	–30.2	CL6156.Contig1, Contig2
rsa-miRn24	0	25	GGTGCAGTTCGGGACTGATTG	5′	21	110	–48.8	FD955742
rsa-miRn24^*^	0	10	ATTGGCTCCCGCCTTGCATCAA	3′	22	110	–48.8	FD955742

*LP (nt), The length of precursor; MFE (kcal/mol), Minimum free energy*.

### Identification of CMS occurrence-related miRNAs during anther development in radish

**Figure 2:**
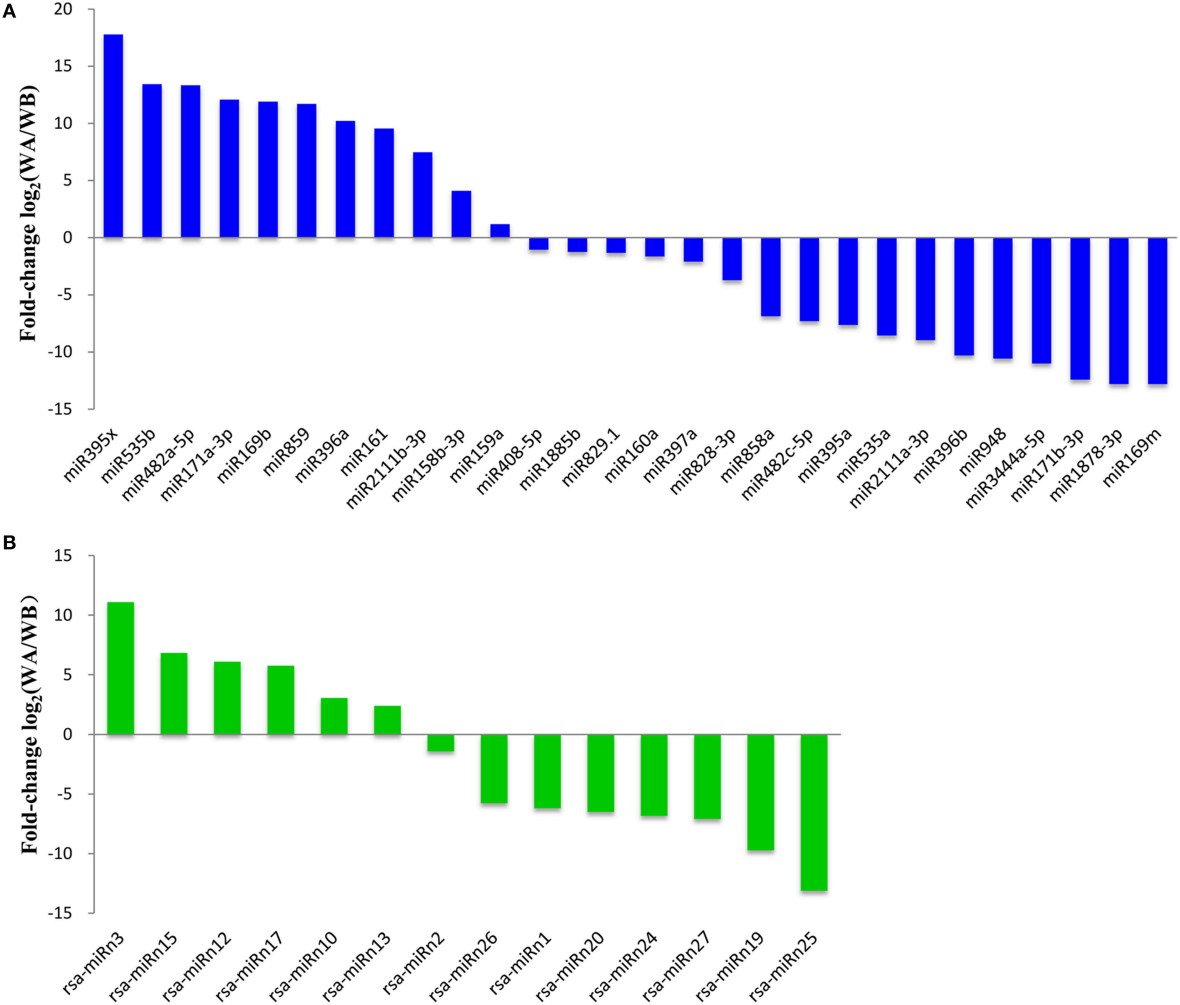
**Comparative relative expression of differentially expressed known (A) and potential novel (B) miRNAs between WA and WB library from radish floral buds**.

To identify miRNAs involved in CMS occurrence during anther development in radish, the differential expression of miRNAs in WA, and WB library was analyzed. Based on these rigorous set of criteria above, a total of 28 known and 14 potential novel miRNAs were differentially expressed during anther development (Figure 2, Table S6). Among them, 17 miRNAs including 11 known and 6 novel ones were up-regulated, and 25 miRNAs including 17 known and 8 novel ones were down-regulated. Of these, 15 miRNAs were differentially expressed at a ratio greater than 10-fold, including 13 known, and two novel miRNAs. Especially, two miRNAs, miR395x (17.77-fold) and rsa-miRn3 (11.09-fold) were the most significantly up-regulated known and novel miRNA, respectively (Figure 2). In addition, many of these CMS occurrence-related miRNAs including miR169m, miR171b-3p, miR396b, miR482c-5p, miR1878-3p, and miR3444a-5p were confined to be expressed only in the WA library, whereas miR171a-3p, miR396a, miR482a-5p, and miR859 were only detected in the WB library. The findings suggested that these miRNAs may play critical roles during anther development in radish.

### Target prediction of CMS occurrence-related miRNAs in radish

**Figure 3:**
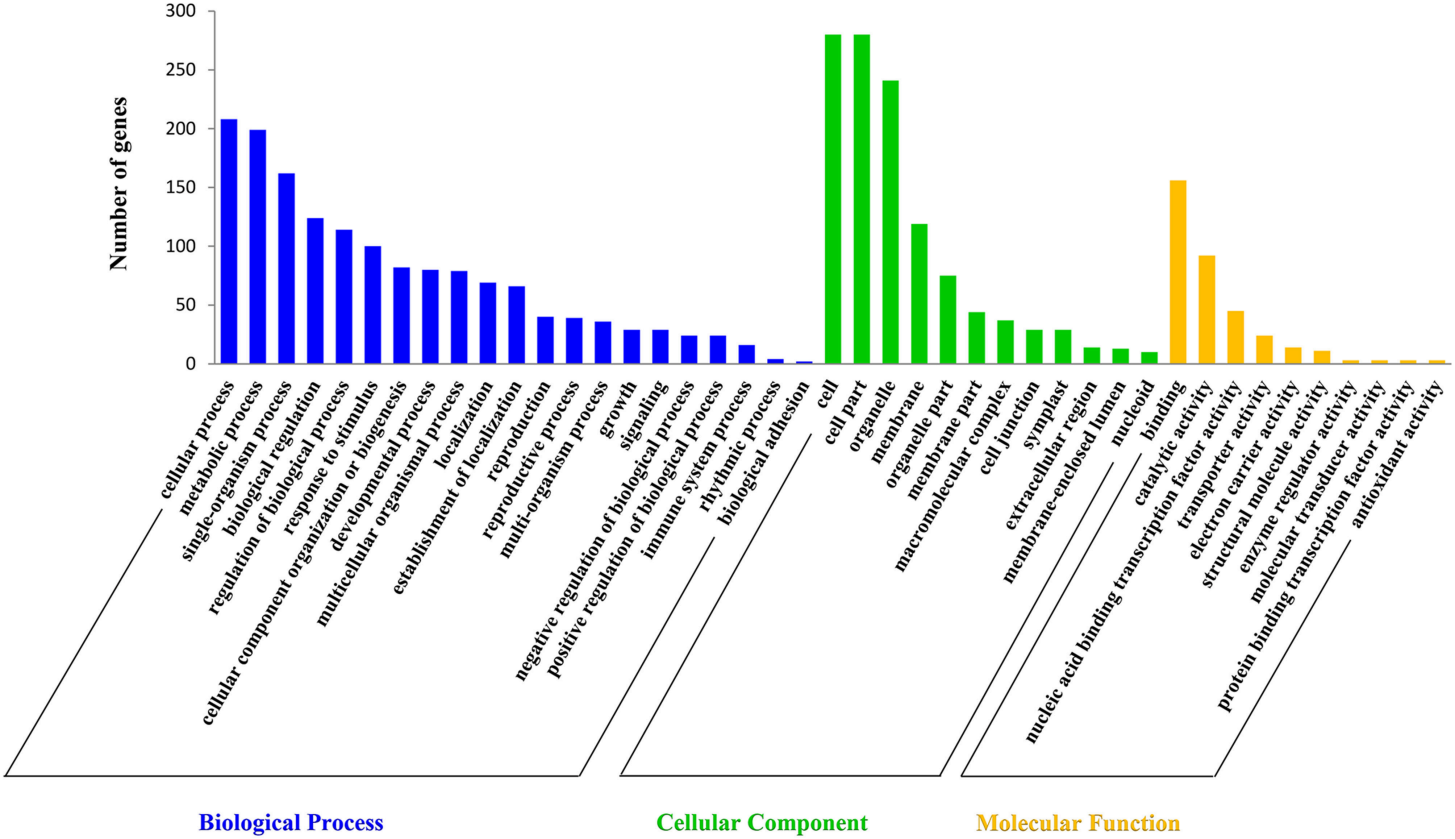
**Gene ontology classification of the predicted targets for differentially expressed miRNAs**.

Target prediction is a prerequisite to understand the biological functions of miRNAs during anther development. In this study, a total of 489 target transcripts were predicted for all the identified miRNAs in radish (Tables S7, S8). To further understand the biological functions of miRNAs, the annotation of these target transcripts were classified into three GO ontologies using the Blast2GO program (http://www.blast2go.com), including 21 biological processes, 12 cellular components, and 10 molecular functions (Figure 3). The main terms in biological processes were “cellular process” (GO: 0009987), “metabolic process” (GO: 0008152), “single-organism process” (GO: 0044699), and “biological regulation” (GO: 0065007). In regard to cellular components, “cell” (GO: 0005623), “cell part” (GO: 0044464), and “organelle” (GO: 0043226) were the three most abundant terms. In addition, “binding” (GO: 0005488) and “catalytic activity” (GO: 0003824) were the most abundant subcategories in the molecular functions.

To understand the biological functions of the isolated miRNAs in radish, the miRNA-cleaved mRNAs during anther development were identified. In this study, 489 potential target sequences for 53 known, 16 potential novel and 84 unclassified non-conserved miRNAs from the transcripts of WA and WB library were further annotated by BLAST search against Arabidopsis sequences using KOBAS 2.0 program (Tables S7, S8). Among these predicted targets, a large proportion of them are known transcription factor families such as auxin response factors (*ARFs*), basic-leucine zippers (*bZIPs*), myb domain proteins (*MYBs*), and squamosa promoter-binding proteins (*SPLs*), which could play essential roles in anther development and CMS occurrence of radish. Moreover, several target genes encoding functional proteins play roles in a broad range of biological processes including agamous-like MADS-box protein 16 (*AGL16*), argonaute (*AGO*), F-box protein (*F-box*), NAC domain containing protein 96 (*NAC096*), pentatricopeptide repeat-containing protein (*PPR*), and protein TRANSPORT INHIBITOR RESPONSE 1 (*TIR1*) (Tables 4, S7). To gain further insight into the correlations between miRNAs and their targets, the miRNA-targets regulatory network was constructed (Figure S5, Table S9). Among them, 26 miRNAs including 19 known and 7 potential novel ones, and 87 unique targets formed a total of 93 miRNA–targets pairs with negatively correlated expression during anther development. In general, these results suggested that the differentially expressed miRNAs may play fundamental regulatory roles in diverse aspects of biological processes during anther development of radish.

**Table 4:** **Identified candidate targets for some known and potential novel miRNAs during anther development**.

**miRNA**	**Target sequence**	**Target gene**	**Target gene annotation**
miR156a	Rsa#S41982434	*CYP705A15*	Cytochrome P450, family 705, subfamily A, polypeptide 15
	Rsa#S43017568	*SPL3*	Squamosa promoter-binding-like protein 3
	CL289.Contig1	*SPL5*	Squamosa promoter-binding-like protein 5
	CL2234.Contig1	*SPL13*	Squamosa promoter-binding-like protein 13
	Unigene3780	*OTP82*	Chloroplast RNA editing factor
miR158b-3p	Rsa#S42049270	*PPR*	Pentatricopeptide repeat-containing protein
miR159a	Rsa#S42037487	*MYB101*	Myb domain protein 101
	Rsa#S41979156	*SPL*	Putative transcription factor SPL
	CL8717.Contig1	*SPL*	Putative transcription factor SPL
miR160a	Rsa#S42581764	*ARF16*	Auxin response factor 16
	Unigene466		Hydroxymethylglutaryl-CoA lyase
miR161	CL1282.Contig1	*MSL10*	Mechanosensitive channel of small conductance-like 10
	Unigene28541		Transcription initiation factor TFIIE alpha subunit
miR169b	CL2169.Contig1		26S proteasome non-ATPase regulatory subunit 14
miR169m	CL8331.Contig1		Sulfite exporter TauE/SafE family protein
miR171a-3p	FD953436		Peroxisomal nicotinamide adenine dinucleotide carrier
	CL271.Contig2	*LTP4*	Non-specific lipid-transfer protein 4
miR393a	FD955493	*TIR1*	Protein TRANSPORT INHIBITOR RESPONSE 1
	Unigene359	*EMB2726*	Elongation factor Ts family protein
miR395a	Unigene14836		Putative F-box/kelch-repeat protein
miR396a	CL879.Contig1		Transducin/WD-40 repeat-containing protein
	CL6202.Contig1	*SIP2;1*	Putative aquaporin SIP2-1
miR396b	Unigene20881	*AGO5*	Argonaute 5
	Unigene22800	*PPR*	Pentatricopeptide repeat-containing protein
miR396b-3p	Rsa#S41989522		Transducin/WD-40 repeat-containing protein
miR397a	CL379.Contig2		D-glycerate 3-kinase
	Unigene14031		Syntaxin/t-SNARE family protein
miR403	Rsa#S41987411	*AGO2*	Argonaute 2
	CL3585.Contig3	*AGO2*	Argonaute 2
miR482c-5p	CL561.Contig2		Serine/threonine protein kinase
	CL561.Contig4		Protein kinase family protein
miR854	Rsa#S42041817		Carboxylate clamp-tetratricopeptide repeat protein HOP2
	Rsa#S41978503	*F-box*	F-box protein
	CL5880.Contig1	*bZIP*	bZIP transcription factor
miR1878-3p	Rsa#S42043459		Putative metal tolerance protein C3
miR1885b	CL9579.Contig1	*CSLE1*	Cellulose synthase-like protein E1
	Unigene1615		Probable 26S proteasome non-ATPase regulatory subunit 3b
miR2111a-3p	CL444.Contig1	*AHL19*	AT-hook motif nuclear-localized protein
miR2111b-3p	CL4779.Contig1	*GLN1.3*	Glutamine synthetase cytosolic isozyme 1-3
miR2199	Rsa#S42571099	*AGL16*	Agamous-like MADS-box protein AGL16
miR3444a-5p	Rsa#S42004586	*PRX Q*	Peroxiredoxin Q
	Rsa#S42563276	*AGP16*	Arabinogalactan protein 16
	Rsa#S43011644	*FLA3*	Fasciclin-like arabinogalactan protein 3
	CL2205.Contig1	*F-box*	F-box protein
rsa-miRn2	Rsa#S42571626		Putative pectate lyase 18
rsa-miRn10	CL6997.Contig1	*GR-RBP2*	Glycine-rich RNA-binding protein 2
	CL7005.Contig1	*ATR1*	NADPH–cytochrome P450 reductase 1
rsa-miRn13	FD957134	*HB20*	Homeobox-leucine zipper protein ATHB-20
rsa-miRn15	Unigene22510	*NAC096*	NAC domain containing protein 96
rsa-miRn16	Unigene25057		Bromo-adjacent homology (BAH) domain-containing protein
rsa-miRn20	CL9688.Contig1	*PKP-BETA1*	Plastidial pyruvate kinase 2
	Unigene18839		Anticodon-binding domain-containing protein

### qRT–PCR validation of miRNAs and their targets during anther development

**Figure 4:**
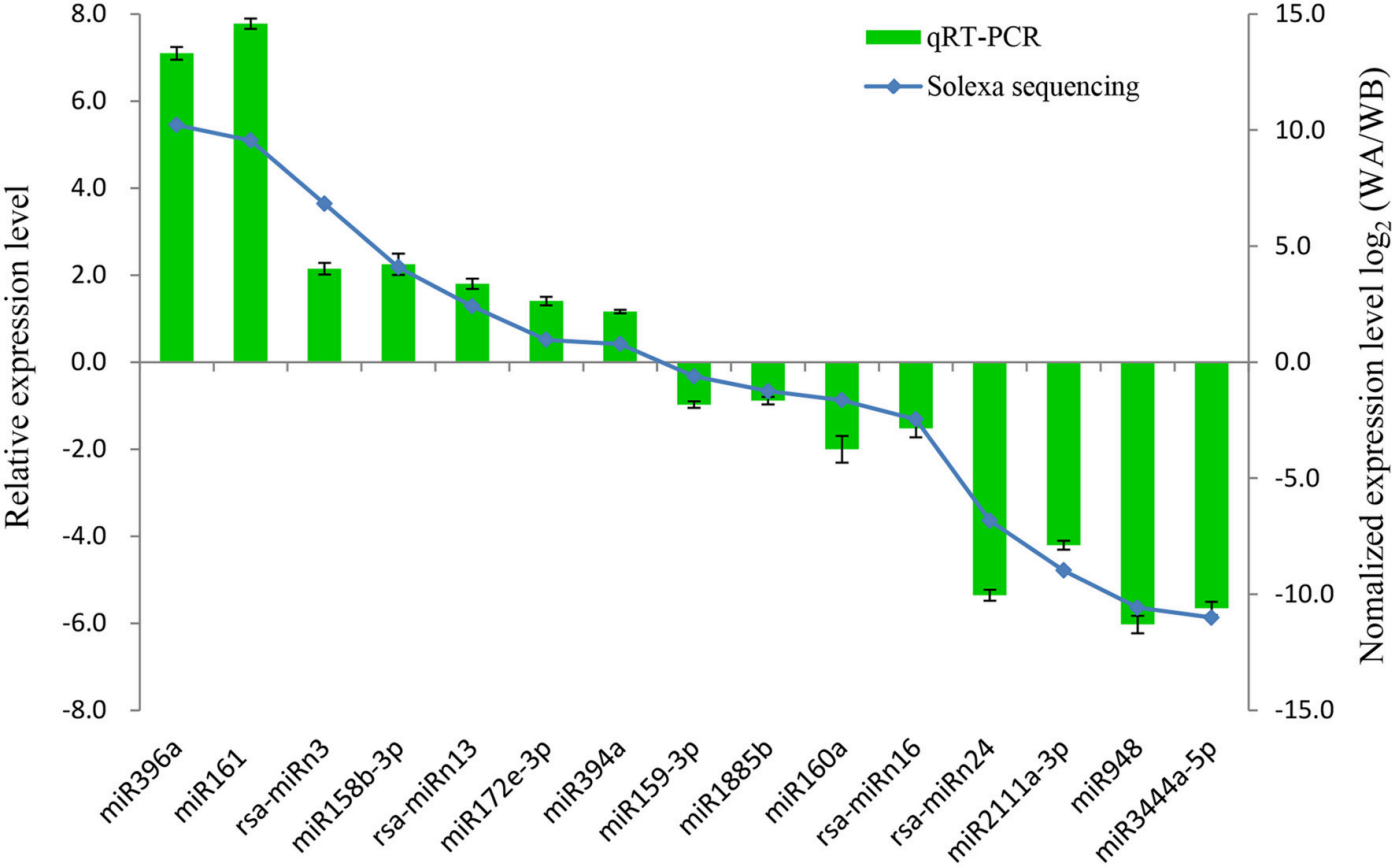
**Comparison of relative expression levels of miRNAs between qRT-PCR and small RNA sequencing in radish**. Data are means ± *SD* from triplicate assays.

**Figure 5:**
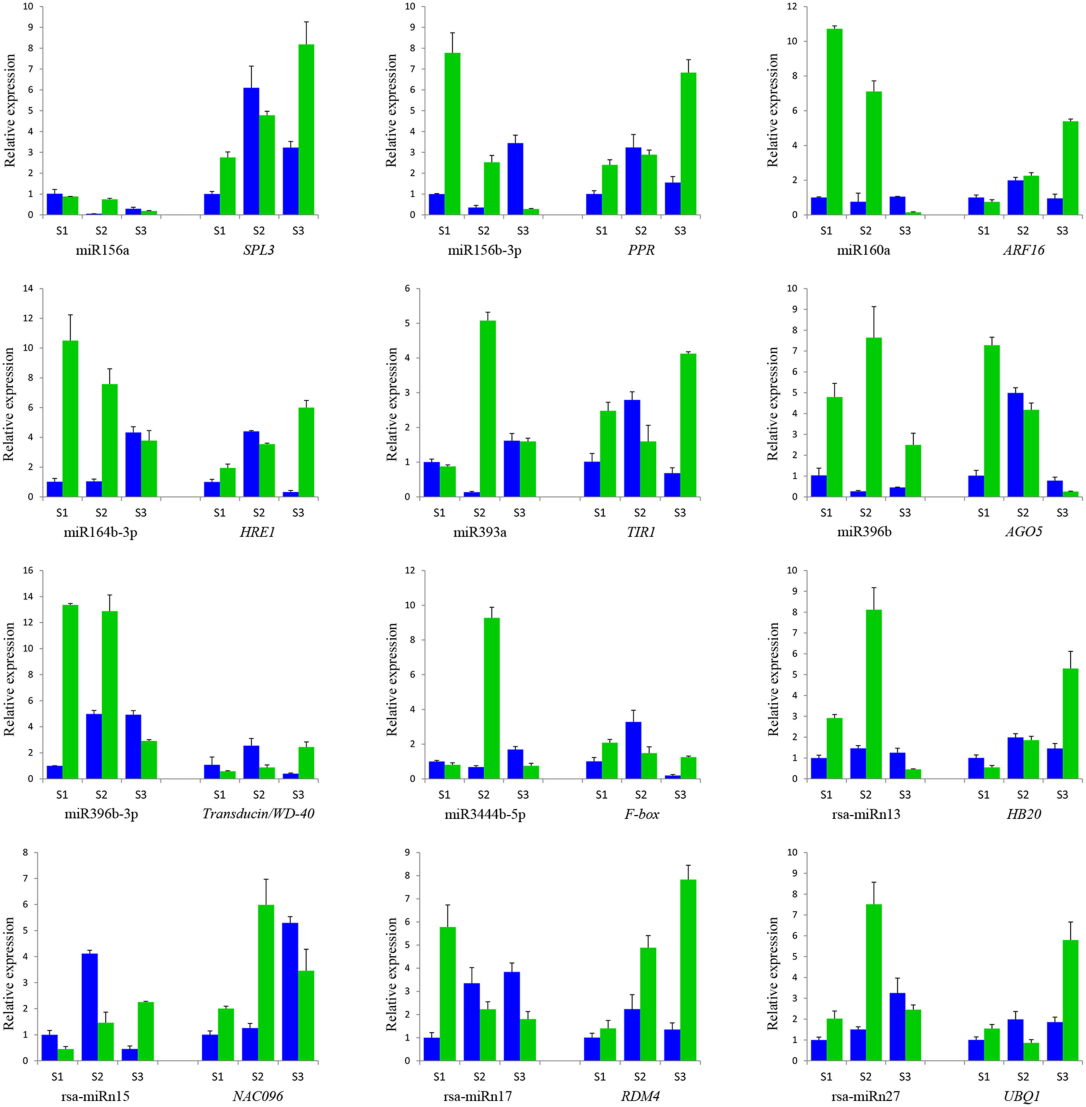
**qRT-PCR validation of differentially expressed miRNAs and its corresponding target genes in floral buds of ‘WA’ (in green) and ‘WB’ (in blue)**. (S1) meiosis stage, (S2) tetrad stage, (S3) early microspore stage. Data are means ± *SD* from triplicate assays.

To verify the quality of small RNA sequencing and analyze the expression patterns of CMS occurrence-related miRNAs in radish, a total of 15 miRNAs were randomly selected for qRT-PCR analysis. It was shown that the expression patterns of these miRNAs from qRT-PCR displayed a similar tendency with those from small RNA sequencing (Figure 4). To further study the dynamic expression patterns of CMS occurrence-related miRNAs and their corresponding targets during anther development, a total of 12 predicted target genes, *SPL3* (Rsa#S43017568 targeted by miR156a), *PPR* (Rsa#S42049270 targeted by miR158b-3p), *ARF16* (Rsa#S42581764 targeted by miR160a), *HRE1* (Rsa#S43010415 targeted by miR164b-3P), *TIR1* (FD955493 targeted by miR393a), *AGO5* (Unigene20881 targeted by miR396b), *Transducin/WD-40* (Rsa#S41989522 targeted by miR396b-3p), *F-box* (CL2205.Contig1 targeted by miR3444a-5p), *HB20* (Rsa#S43028702 targeted by rsa-miRn13), *NAC096* (Unigene22510 targeted by rsa-miRn15), *RDM4* (CL8993.Contig1 targeted by rsa-miRn17), and *UBQ1* (Rsa#S42012413 targeted by rsa-miRn27), were examined by qRT-PCR at three different stages of meiosis, tetrad, and early microspore. As shown in Figure 5, miR158b-3p, miR160a, miR164b-3p, and miR396b-3p were up-regulated and the expression levels maximized at meiosis stage, and then decreased at tetrad and early microspore stage. In addition, miR156a, miR393a, and miR3444a-5p were down-regulated at meiosis stage, and the expression levels then peaked at tetrad stage, but rapidly decreased at early microspore stage. miR396b showed an up-regulated expression pattern and peaked at tetrad stage, and then slightly decreased at early microspore stage. For the novel miRNAs, the expression levels of rsa-miRn13 and rsa-miRn27 were up-regulated at meiosis and tetrad stage, but dramatically decreased to the minimum at early microspore stage. Moreover, rsa-miRn15 was down-regulated at meiosis and tetrad stage, but rapidly increased to the maximum at early microspore stage. Transcripts of rsa-miRn17 reached its maximum at meiosis stage, but sharply declined at tetrad and early microspore stage. Furthermore, some negative correlations could be found between the expression levels of miRNAs and their corresponding target genes during various anther development stages, suggesting that miRNA-mediated mRNA silencing may be involved in CMS occurrence during anther development in ‘WA’ and ‘WB’ line (Figure 5).

## Discussion

High-throughput sequencing technology helps identify a large number of miRNAs and targets associated with CMS occurrence during anther development in several plant species (Wei et al., 2011, 2013; Fang et al., 2014; Yan et al., 2015), and provide an effective way to evaluate the expression profiles of miRNAs and targets in different tissues at different developmental stages. The production of functional pollen grain is a prerequisite for the propagation in flowering plants, and the tapetum cell plays a critical role in microspore and pollen formation (Goetz et al., 2001). Unlike the radish CMS line ‘WA’ having no pollen in aborted anthers, its maintainer line ‘WB’ has normal anthers with fertile pollen (Figure S1). Cytological studies show that there is no visible difference between these two lines during the meiosis and tetrad stage (Figure S1). Thereafter, as compared with ‘WB’, the expanded, and vacuolated tapetum cells of ‘WA’ resulted in microspore degeneration and finally aborted anther with no pollen grains (Figure S1). However, few studies on the relationships between miRNAs and CMS occurrence during anther development in radish were conducted. The lack of CMS occurrence-related genes seriously hampered our understanding of molecular mechanism in CMS occurrence, which became an obstacle to utilize the heterosis of radish. To uncover the miRNA-mediated regulatory network of CMS occurrence during anther development, a comparative small RNAome sequencing was conducted in ‘WA’ and ‘WB’ line in this study. To our current knowledge, this study is the first investigation on identification and characterization of miRNAs, and their targets during anther development in radish.

With the application of high-throughput sequencing technology, it has provided an efficient tool to identify a quite comprehensive set of miRNAs at different stages and to reveal the miRNA-mediated regulatory network of CMS occurrence during anther development in plant. In this study, the length distribution of sRNAs suggested that the 24 nt sRNAs were the most abundant, followed by 21 nt sRNAs, which has been reported in *Arabidopsis* (Voinnet, 2009), *Prunus mume* (Gao et al., 2012), *O. sativa* (Ma et al., 2013), and *Medicago truncatula* (Eyles et al., 2013). The whole frequent percent of 21 and 24 nt small RNAs (28.33 and 30.07%, respectively) in radish was strikingly different from that of *B. juncea*, which 21 nt RNAs had high abundance (> 95%), and 24 nt RNAs possessed low frequency (1.1%) (Yang et al., 2013). Interestingly, the same tendency also existed when compared with *B. rapa* in which 24 nt sRNAs were the most dominant, followed by 21, 22, and 23 nt small RNAs (Jiang et al., 2014), it could be speculated that the genetic relationship between radish and *B. rapa* is closer than that between radish and *B. juncea* in the process of evolution.

Identification a set of miRNAs is a crucial step to promote our understanding of miRNA-mediated regulatory network of anther development and CMS occurrence. Recently, numerous studies have presented that the majority of known miRNAs in plantae are evolutionarily conserved (Chen et al., 2012; Barvkar et al., 2013). The diversity of known miRNA families in radish might be decided by the abundance and number of members. In the present study, a large number of conserved miRNAs expressed relatively higher levels compared with non-conserved ones, which was in agreement with previous researches in other species (Gao et al., 2012; Wang F. D. et al., 2012; Wang Z. J. et al., 2012; Fang et al., 2014). In addition, several studies have reported a number of known and potential novel miRNAs involved in anther development and CMS occurrence in *B. juncea* (Yang et al., 2013), *B. rapa* (Jiang et al., 2014), *Citrus reticulata* (Fang et al., 2014), *G. hirsutum* (Wei et al., 2013), and *O. sativa* (Yan et al., 2015), which greatly enhanced our knowledge of the regulatory roles of miRNAs in CMS occurrence. In this study, 28 known miRNAs were differentially expressed and the majority of these miRNAs were down-regulated during anther development. The differential expression patterns of rsa-miR160a and rsa-miR169b were consistent with those observed in *O. sativa* (Yan et al., 2015). Moreover, the expression pattern of rsa-miR396b and rsa-miR171a-3p was similar to that identified in *G. hirsutum* and *B. rapa*, respectively (Wei et al., 2013; Jiang et al., 2014). Interestingly, the targets of the miR160 contain three critical regulators, *ARF10, ARF16*, and *ARF17*, which are important in mediating gene expression response to the plant hormone auxin and regulating floral organ formation (Mallory et al., 2005; Wang et al., 2005; Chapman and Estelle, 2009; Liu et al., 2010). The expression level of rsa-miR160a was down-regulated in ‘WA’ and validated by qRT-PCR (Figures 5, S5). Thus, it could be speculated that the decreased abundance of rsa-miR160a may partially increase the expression of *ARF16*, finally resulting in abnormal pollen development in the sterile line ‘WA’.

**Figure 6:**
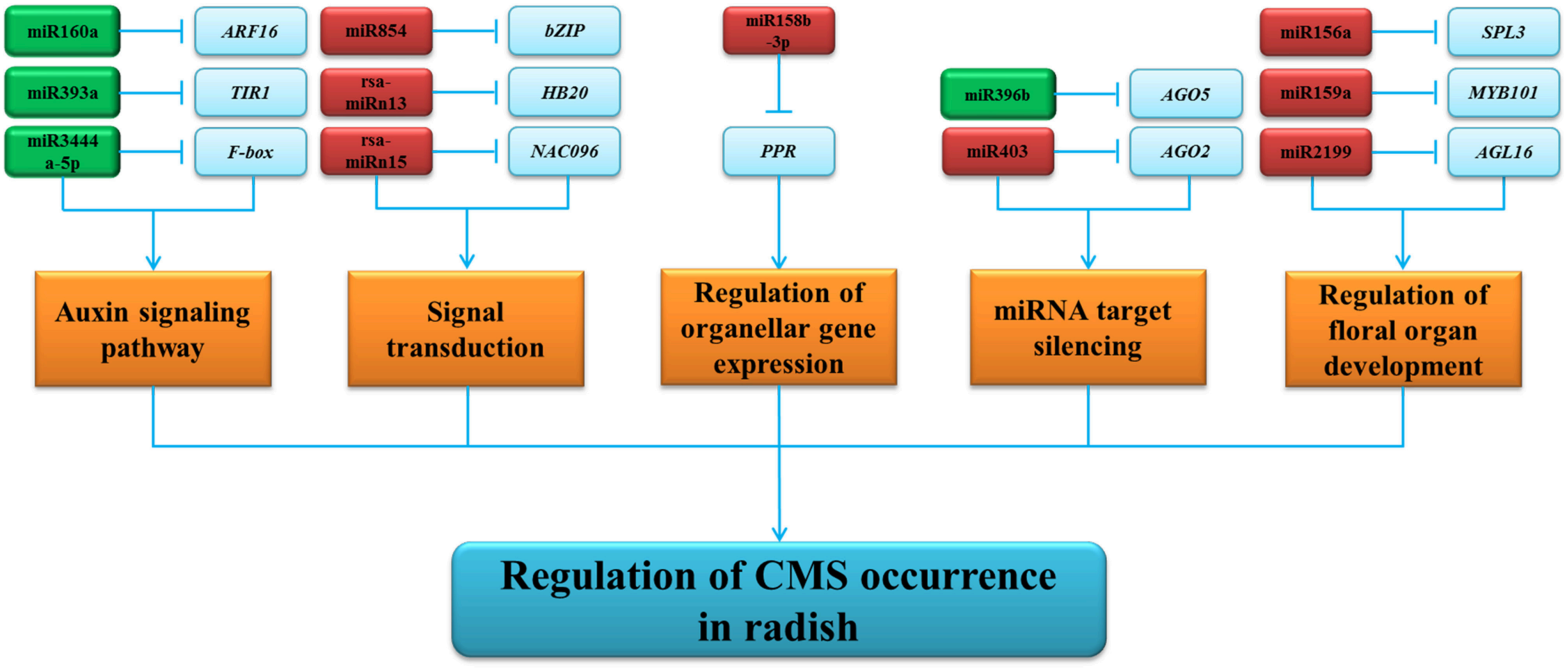
**The hypothetical schematic model of miRNA-mediated regulatory network of CMS occurrence during anther development in radish**. The up- and down-regulated miRNAs are in red and green, respectively. Agamous-like MADS-box protein AGL16 (*AGL16*), argonaute 2 (*AGO2*), argonaute 5 (*AGO5*), auxin response factor 16 (*ARF16*), basic leucine-zipper (*bZIP*), F-box protein (*F-box*), myb domain protein 101 (*MYB101*), NAC domain containing protein 96 (*NAC096*), pentatricopeptide repeat-containing protein (*PPR*), protein TRANSPORT INHIBITOR RESPONSE 1 (*TIR1*), squamosa promoter-binding like protein 3 (*SPL3*).

According to the negative correlation between differentially expressed miRNAs and their corresponding targets (Figure S5), a hypothetical schematic model of miRNA-mediated regulatory network of CMS occurrence during anther development in radish was put forward (Figure 6). As shown in the regulatory network, targets of these differentially expressed miRNAs containing important transcription factors (TFs) and functional proteins are involved in many biological processes, including auxin signaling pathways, signal transduction, miRNA target silencing, floral organ development, and organellar gene expression. For instance, SBP-box genes targeted by miR156, a group of TFs with significant regulatory functions controlling the transition from the vegetative phase to the floral phase in *Arabidopsis, O. sativa*, and *Zea mays* (Chuck et al., 2007; Gandikota et al., 2007; Jiao et al., 2010). It was reported that three genes, *LEAFY, FRUITFULL*, and *APETALA1*, are directly activated by *SPL3* to regulate the timing of flower formation (Yamaguchi et al., 2009). Additionally, multiple SPL genes can lead to fully fertile flowers and regulate cell division and differentiation in *Arabidopsis* (Xing et al., 2010). In the present study, up-regulation of the rsa-miR156a decreased the expression of *SPL3* in ‘WA’ compared to ‘WB’ (Figure 6, Table S7), leading to disordered floral organ development, cell division, and differentiation in radish. MiR159 is required for normal anther development, in which it regulates the expression of genes encoding MYB TFs (Achard et al., 2004; Tsuji et al., 2006). MYB TFs are involved in the control of plant development, determination of cell fate and identity, primary, and secondary metabolism (Stracke et al., 2007; Gonzalez et al., 2008; Kang et al., 2009). *AtMYB103*, specifically expressed in tapetums and middle layers of anthers, is important for pollen development, especially the pollen exine formation (Zhang et al., 2007; Chen et al., 2014). Down-regulation of the *AtMYB103* resulted in earlier degeneration of tapetum and pollen grains aberration during anther development in *A. thaliana* (Zhang et al., 2007). In rice, anther and pollen defect in floral organ development are also found in the loss-of-function mutations of MYB (Kaneko et al., 2004). In the present study, the rsa-miR159a was found to be up-regulated in ‘WA’ line compared to ‘WB’ line (Figures 6, S5), indicating that the increased abundance of rsa-miR159a partially decreased the expression of *MYB101*, hampering normal tapetum development, callose dissolution, and exine formation in radish anthers. Moreover, *AGL16*, belonging to MADS-box transcription factors, was identified to be targeted by rsa-miR2199. The MADS-box TFs are essential regulators of the development of the floral meristems and floral organs in plants (Chen et al., 2014). These evidences indicated that rsa-miR2199 might be an essential component of gene regulatory network that involved in radish CMS occurrence (Figure 6).

Apart from key TFs, a variety of genes which encode important functional proteins, such as PPR proteins, F-box proteins, AGO proteins, and protein TRANSPORT INHIBITOR RESPONSE 1 (TIR1), were also considered to play important roles in CMS occurrence during anther development. PPR protein genes were identified as targets of miR158 (Lurin et al., 2004; Sunkar and Zhu, 2004). Previous studies indicated that PPR proteins are mostly located in the mitochondria and chloroplast and play crucial roles in pollen development, specific RNA sequence binding, post-transcriptional splicing and mRNA stability regulating (Okuda et al., 2006; Wang et al., 2006; Saze and Kakutani, 2007; Fujii and Small, 2011). In addition, some PPR proteins have also been identified as fertility-restoring genes (*Rf*) for CMS occurrence (Desloire et al., 2003; Wang et al., 2008; Yasumoto et al., 2009). In this study, rsa-miR158b-3p targeting the gene encoding PPR protein was up-regulated and the *PPR* gene was suppressed in ‘WA’ line compared with ‘WB’ line, and it could be suggested that the regular expression of CMS-associated mitochondrial genes and suppression of *PPR* gene result in sterility in radish ‘WA’ line (Figures 5, S5). Moreover, F-box proteins are involved in the regulation of various developmental processes in plants, including floral meristem, floral organ identity determination, and photomorphogenesis (Jain et al., 2007). The expression of rsa-miR3444a-5p was down-regulated at meiosis stage, and then peaked at tetrad stage, but rapidly decreased at early microspore stage, and a negative correlation was found between the expression levels of rsa-miR3444a-5p and its target gene which encoding F-box protein at three different stages according to the qRT-PCR analysis (Figure 5). In addition, *F-box* gene targeted by osa-miR528 was found to be involved in the regulation of the abortion process in male sterile line of rice. Moreover, the other 23 genes including *APG2, AGP16, FIO1, FLA3, FLA5, NAC083, NSP5, TRP1*, and *VIP1* were also the targets of rsa-miR3444a-5p, indicating that the miRNA has multiple effects on the targets (Figure S5). All of these genes targeted by rsa-miR3444a-5p might function together to regulate the CMS occurrence during anther development in radish. Additionally, AGO proteins were reported to be involved in diverse biological processes including hormone response, developmental regulation, and stress adaptation (Yang et al., 2013). Up-regulation of *TIR1* enhances auxin sensitivity, and causes altered leave phenotype and delayed flowering (Chen et al., 2011). In this study, *AGO2* and *AGO5* was targeted by miR403 and miR396b, respectively, and *TIR1* was targeted by miR393a, indicating miR403, miR396b, and miR393a might modulate the hormone response to play roles in the microspore development and CMS occurrence.

In summary, CMS occurrence-associated miRNAs and their targets between the male sterile line ‘WA’ and its maintainer line ‘WB’ were firstly identified and characterized in radish. These results provide a valuable foundation for unraveling the complex miRNA-mediated regulatory network of CMS occurrence and facilitate further dissection of roles of miRNAs during CMS occurrence and microspore formation in radish and other crops.

## Author contributions

WZ, YX, and LL designed the research. WZ and YX conducted experiments. LX and YW participated in the design of the study and performed the statistical analysis. WZ and YX analyzed data and wrote the manuscript. XZ, RW, YZ, and EM helped with the revision of manuscript. All authors read and approved the manuscript.

### Conflict of interest statement

The authors declare that the research was conducted in the absence of any commercial or financial relationships that could be construed as a potential conflict of interest.

## Supplementary material

Figure S1 **Micrographs of anthers at different developmental stages in the CMS line ‘WA’ (A–E) and its maintainer line ‘WB’ (F–J)**. Panels **(A,F)** Meiosis stage. Panels **(B,G)** Tetrad stage. Panels **(C,H)** Early microspore stage. Panels **(D,I)** Pollen stage. Panels **(E,J)** Flower morphology.

Figure S2 **Distribution of known miRNA family members identified in radish**.

Figure S3 **Abundance of each known miRNA family in radish**.

Figure S4 **Precursor sequences and the predicted second structures of novel miRNAs in radish**. The mature miRNAs are in red and miRNA^*^s are in blue (“.” represent base mismatches, “(” represent base matches).

Figure S5 **The miRNA mediated regulatory network constructed by Cytoscape_v3.2.1**. The red, yellow and green ellipses represent the know miRNAs, potential novel miRNAs and target genes, respectively.

Table S1 **Primers of miRNAs and targets in radish for qRT-PCR**.

Table S2 **Statistical analysis of sequencing reads from the WA and WB sRNA library in radish**.

Table S3 **Summary of common and specific sequences between WA and WB sRNA library**.

Table S4 **Detailed information of known miRNAs identified from radish WA and WB library**.

Table S5 **Detailed information of novel miRNAs identified from radish WA and WB library**.

Table S6 **Differentially-expressed miRNAs between WA and WB in radish**.

Table S7 **Putative targets of known and novel miRNAs identified in radish**.

Table S8 **Predicted targets for non-conserved miRNAs in radish**.

Table S9 **The detailed information of miRNA-targets for regulatory network construction**.

## References

[B1] Achard P. Herr A. Baulcombe D. C. Harberd N. P. (2004). Modulation of floral development by a gibberellin-regulated microRNA. Development 131, 3357–3365. 10.1242/dev.01206 15226253

[B2] Allen E. Xie Z. Gustafson A. M. Carrington J. C. (2005). microRNA-directed phasing during trans-acting siRNA biogenesis in plants. Cell 121, 207–221. 10.1016/j.cell.2005.04.004 15851028

[B3] Barvkar V. T. Pardeshi V. C. Kale S. M. Qiu S. Rollins M. Datla R.. (2013). Genome-wide identification and characterization of microRNA genes and their targets in flax (*Linum usitatissimum*). Planta 237, 1149–1161. 10.1007/s00425-012-1833-5 23291876

[B4] Bentolila S. Alfonso A. A. Hanson M. R. (2002). A pentatricopeptide repeat-containing gene restores fertility to cytoplasmic male-sterile plants. Proc. Natl. Acad. Sci. U.S.A. 99, 10887–10892. 10.1073/pnas.102301599 12136123 PMC125068

[B5] Bodersen P. Sakvarelidze-Achard L. Bruun-Rasmussen M. Dunoyer P. Yamamoto Y. Y. Sieburth L.. (2008). Widespread translational inhibition by plant miRNAs and siRNAs. Science 320, 1185–1190. 10.1126/science.1159151 18483398

[B6] Brown G. G. Formanova N. Jin H. Wargachuk R. Dendy C. Patil P.. (2003). The radish Rfo restorer gene of Ogura cytoplasmic male sterility encodes a protein with multiple pentatricopeptide repeats. Plant J. 35, 262–272. 10.1046/j.1365-313X.2003.01799.x 12848830

[B7] Chambers C. Shuai B. (2009). Profiling microRNA expression in Arabidopsis pollen using microRNA array and real-time PCR. BMC Plant Biol. 9:87. 10.1186/1471-2229-9-87 19591667 PMC2715406

[B8] Chapman E. J. Estelle M. (2009). Mechanism of auxin-regulated gene expression in plants. Annu. Rev. Genet. 43, 265–285. 10.1146/annurev-genet-102108-134148 19686081

[B9] Chen L. Liu Y. G. (2014). Male sterility and fertility restoration in crops. Annu. Rev. Plant Biol. 65, 579–606. 10.1146/annurev-arplant-050213-040119 24313845

[B10] Chen L. Ren Y. Zhang Y. Xu J. Sun F. Zhang Z.. (2012). Genome-wide identification and expression analysis of heat-responsive and novel microRNAs in *Populus tomentosa*. Gene 504, 160–165. 10.1016/j.gene.2012.05.034 22634103

[B11] Chen P. Ran S. M. Li R. Huang Z. P. Qian J. H. Yu M. L. (2014). Transcriptome de novo assembly and differentially expressed genes related to cytoplasmic male sterility in kenaf (*Hibiscus cannabinus* L.). Mol. Breed. 34, 1879–1891. 10.1007/s11105-014-0702-8

[B12] Chen Z. H. Bao M. L. Sun Y. Z. Yang Y. J. Xu X. H. Wang J. H.. (2011). Regulation of auxin response by miR393-targeted transport inhibitor response protein 1 is involved in normal development in Arabidopsis. Plant Mol. Biol. 77, 619–629. 10.1007/s11103-011-9838-1 22042293

[B13] Chuck G. Cigan A. M. Saeteurn K. Hake S. (2007). The heterochronic maize mutant Corngrass1 results from overexpression of a tandem microRNA. Nat. Genet. 39, 544–549. 10.1038/ng2001 17369828

[B14] Dai X. Zhao P. X. (2011). psRNATarget: a plant small RNA target analysis server. Nucleic Acids Res. 39, W155–W159. 10.1093/nar/gkr319 21622958 PMC3125753

[B15] Desloire S. Gherbi H. Laloui W. Marhadour S. Clouet V. Cattolico L.. (2003). Identification of the fertility restoration locus, Rfo, in radish, as a member of the pentatricopeptide-repeat protein family. EMBO Rep. 4, 588–594. 10.1038/sj.embor.embor848 12740605 PMC1319198

[B16] Eckardt N. A. (2006). Cytoplasmic male sterility and fertility restoration. Plant Cell 18, 515–517. 10.1105/tpc.106.041830

[B17] Eyles R. P. Williams P. H. Ohms S. J. Weiller G. F. Ogilvie H. A. Djordjevic M. A.. (2013). microRNA profiling of root tissues and root forming explant cultures in *Medicago truncatula*. Planta 238, 91–105. 10.1007/s00425-013-1871-7 23572382

[B18] Fang Y. N. Qiu W. M. Wang Y. Wu X. M. Xu Q. Guo W. W. (2014). Identification of differentially expressed microRNAs from a male sterile *Ponkan mandarin* (*Citrus reticulata* Blanco) and its fertile wild type by small RNA and degradome sequencing. Tree Genet. Genome 10, 1567–1581. 10.1007/s11295-014-0780-7

[B19] Fujii S. Small I. (2011). The evolution of RNA editing and pentatricopeptide repeat genes. New Phytol. 191, 37–47. 10.1111/j.1469-8137.2011.03746.x 21557747

[B20] Gandikota M. Birkenbihl R. P. Höhmann S. Cardon G. H. Saedler H. Huijser P. (2007). The miRNA156/157 recognition element in the 3′UTR of the Arabidopsis SBP box gene *SPL3* prevents early flowering by translational inhibition in seedlings. Plant J. 49, 683–693. 10.1111/j.1365-313X.2006.02983.x 17217458

[B21] Gao Z. H. Shi T. Luo X. Y. Zhang Z. Zhuang W. B. Wang L. J. (2012). High-throughput sequencing of small RNAs and analysis of differentially expressed microRNAs associated with pistil development in Japanese apricot. BMC Genomics 13:371. 10.1186/1471-2164-13-371 22863067 PMC3464595

[B22] Goetz M. Godt D. E. Guivarc'h A. Kahmann U. Chriqui D. Roitsch T. (2001). Induction of male sterility in plants by metabolic engineering of the carbohydrate supply. Proc. Natl. Acad. Sci. U.S.A. 98, 6522–6527. 10.1073/pnas.091097998 11371651 PMC33501

[B23] Gonzalez A. Zhao M. Leavitt J. M. Lloyd A. M. (2008). Regulation of the anthocyanin biosynthetic pathway by the TTG1/bHLH/Myb transcriptional complex in Arabidopsis seedlings. Plant J. 53, 814–827. 10.1111/j.1365-313X.2007.03373.x 18036197

[B24] Hafner M. Landgraf P. Ludwig J. Rice A. Ojo T. Lin C.. (2008). Identification of microRNAs and other small regulatory RNAs using cDNA library sequencing. Methods 44, 3–12. 10.1016/j.ymeth.2007.09.009 18158127 PMC2847350

[B25] Henderson I. R. Zhang X. Lu C. Johnson L. Meyers B. C. Green P. J.. (2006). Dissecting *Arabidopsis thaliana* DICER function in small RNA processing, gene silencing and DNA methylation patterning. Nat. Genet. 38, 721–725. 10.1038/ng1804 16699516

[B26] Hu J. Wang K. Huang W. C. Liu G. Gao Y. Wang J. M.. (2012). The rice pentatricopeptide repeat protein RF5 restores fertility in Hong-Lian cytoplasmic male-sterile lines via a complex with the glycine-rich protein GRP162. Plant Cell 24, 109–122. 10.1105/tpc.111.093211 22247252 PMC3289560

[B27] Jain M. Nijhawan A. Arora R. Agarwal P. Ray S. Sharma P.. (2007). F-box proteins in rice. Genome-wide analysis, classification, temporal and spatial gene expression during panicle and seed development, and regulation by light and abiotic stress. Plant Physiol. 143, 1467–1483. 10.1104/pp.106.091900 17293439 PMC1851844

[B28] Jiang J. X. Lv M. L. Liang Y. Ma Z. M. Cao J. S. (2014). Identification of novel and conserved miRNAs involved in pollen development in *Brassica campestris* ssp. *chinensis* by high-throughput sequencing and degradome analysis. BMC Genomics 15:146. 10.1186/1471-2164-15-146 24559317 PMC3936892

[B29] Jiao Y. Q. Wang Y. H. Xue D. W. Wang J. Yan M. X. Liu G. F. (2010). Regulation of *OsSPL14* by OsmiR156 defines ideal plant architecture in rice. Nat. Genet. 42, 541–544. 10.1038/ng.591 20495565

[B30] Jones-Rhoades M. W. Bartel D. P. Bartel B. (2006). MicroRNAs and their regulatory roles in plants. Annu. Rev. Plant Biol. 57, 19–53. 10.1146/annurev.arplant.57.032905.105218 16669754

[B31] Kaneko M. Inukai Y. Ueguchi-Tanaka M. Itoh H. Izawa T. Kobayashi Y.. (2004). Loss-of-function mutations of the rice *GAMYB* gene impair α-amylase expression in aleurone and flower development. Plant Cell 16, 33–44. 10.1105/tpc.017327 14688295 PMC301393

[B32] Kang Y. H. Kirik V. Hulskamp M. Nam K. H. Hagely K. Lee M. M.. (2009). The *MYB23* gene provides a positive feedback loop for cell fate specification in the Arabidopsis root epidermis. Plant Cell 21, 1080–1094 10.1105/tpc.108.063180 19395683 PMC2685616

[B33] Kubo T. Kitazaki K. Matsunaga M. Kagami H. Mikami T. (2011). Male sterility-inducing mitochondrial genomes: how do they differ? Crit. Rev. Plant Sci. 30, 378–400. 10.1080/07352689.2011.587727

[B34] Kurihara Y. Takashi Y. Watanabe Y. (2006). The interaction between DCL1 and HYL1 is important for efficient and precise processing of pri-miRNA in plant microRNA biogenesis. RNA 12, 206–212. 10.1261/rna.2146906 16428603 PMC1370900

[B35] Li R. Q. Yu C. Li Y. R. Lam T. W. Yiu S. M. Kristiansen K.. (2009). SOAP2: an improved ultrafast tool for short read alignment. Bioinformatics 25, 1966–1967. 10.1093/bioinformatics/btp336 19497933

[B37] Liu X. D. Huang J. Wang Y. Khanna K. Xie Z. X. Owen H. A.. (2010). The role of floral organs in carpels, an Arabidopsis loss-of-function mutation in MicroRNA160a, in organogenesis and the mechanism regulating its expression. Plant J. 62, 416–428. 10.1111/j.1365-313X.2010.04164.x 20136729

[B36] Livak K. J. Schmittgen T. D. (2001). Analysis of relative gene expression data using real-time quantitative PCR and the 2^−ΔΔ*C*_T_^ method. Methods 25, 402–408. 10.1006/meth.2001.1262 11846609

[B38] Lurin C. Andrés C. Aubourg S. Bellaoui M. Bitton F. Bruyère C.. (2004). Genome-wide analysis of Arabidopsis pentatricopeptide repeat proteins reveals their essential role in organelle biogenesis. Plant Cell 16, 2089–2103. 10.1105/tpc.104.022236 15269332 PMC519200

[B39] Ma X. X. Shao C. G. Wang H. Z. Jin Y. F. Meng Y. J. (2013). Construction of small RNA-mediated gene regulatory networks in the roots of rice (*Oryza sativa*). BMC Genomics 14:510. 10.1186/1471-2164-14-510 23889819 PMC3734165

[B40] Mallory A. C. Bartel D. P. Bartel B. (2005). MicroRNA-directed regulation of Arabidopsis *AUXIN RESPONSE FACTOR17* is essential for proper development and modulates expression of early auxin response genes. Plant Cell 17, 1360–1375. 10.1105/tpc.105.031716 15829600 PMC1091760

[B41] Meyers B. C. Axtell M. J. Bartel B. Bartel D. P. Baulcombe D. Bowman J. L.. (2008). Criteria for annotation of plant MicroRNAs. Plant Cell 20, 3186–3190. 10.1105/tpc.108.064311 19074682 PMC2630443

[B42] Nie S. S. Xu L. Wang Y. Huang D. Q. Muleke E. M. Sun X. C.. (2015). Identification of bolting-related microRNAs and their targets reveals complex miRNA-mediated flowering-time regulatory networks in radish (*Raphanus sativus* L.). Sci. Rep. 5:14034. 10.1038/srep14034 26369897 PMC4570191

[B43] Okuda K. Nakamura T. Sugita M. Shimizu T. Shikanai T. (2006). A pentatricopeptide repeat protein is a site recognition factor in chloroplast RNA editing. J. Biol. Chem. 28, 37661–37667. 10.1074/jbc.M608184200 17015439

[B44] Rajagopalan R. Vaucheret H. Trejo J. Bartel D. P. (2006). A diverse and evolutionarily fluid set of microRNAs in *Arabidopsis thaliana*. Genes Dev. 20, 3407–3425. 10.1101/gad.1476406 17182867 PMC1698448

[B45] Ruiz-Ferrer V. Voinnet O. (2009). Roles of plant small RNAs in biotic stress responses. Annu. Rev. Plant Biol. 60, 485–510. 10.1146/annurev.arplant.043008.092111 19519217

[B46] Saze H. Kakutani T. (2007). Heritable epigenetic mutation of a transposon-flanked Arabidopsis gene due to lack of the chromatin-remodeling factor DDM1. EMBO J. 26, 3641–3652. 10.1038/sj.emboj.7601788 17627280 PMC1949009

[B47] Schnable P. S. Wise R. P. (1998). The molecular basis of cytoplasmic male sterility and fertility restoration. Trends Plant Sci. 3, 175–180. 10.1016/S1360-1385(98)01235-7

[B48] Smoot M. E. Ono K. Ruscheinski J. Wang P. L. Ideker T. (2011). Cytoscape 2.8: new features for data integration and network visualization. Bioinformatics 27, 431–432. 10.1093/bioinformatics/btq675 21149340 PMC3031041

[B49] Stracke R. Ishihara H. Huep G. Barsch A. Mehrtens F. Niehaus K.. (2007). Differential regulation of closely related R2R3-MYB transcription factors controls flavonol accumulation in different parts of the *Arabidopsis thaliana* seedling. Plant J. 50, 660–677. 10.1111/j.1365-313X.2007.03078.x 17419845 PMC1976380

[B50] Sun X. J. Liu Y. Wang L. J. Zhu X. W. Gong Y. Q. Xu L. (2012). Molecular characterization of the *Rs-Rf1* gene and molecular marker-assisted development of elite radish (*Raphanus sativus* L.) CMS lines with a functional marker for fertility restoration. Mol. Breeding 30, 1727–1736. 10.1007/s11032-012-9756-1

[B51] Sun X. J. Wang Y. Chen Y. L. Xu L. Jiang L. N. Gong Y. Q. (2013). Differential gene expression profiling of Ogura CMS line and its maintainer in radish (*Raphanus sativus* L.). Acta Physiol. Plant. 35, 3413–3425. 10.1007/s11738-013-1376-9

[B52] Sunkar R. Zhu J. K. (2004). Novel and stress-regulated microRNAs and other small RNAs from Arabidopsis. Plant Cell 16, 2001–2019. 10.1105/tpc.104.022830 15258262 PMC519194

[B53] Touzet P. Meyer E. H. (2014). Cytoplasmic male sterility and mitochondrial metabolism in plants. Mitochondrion 19, 166–171. 10.1016/j.mito.2014.04.009 24769053

[B54] Tsuji H. Aya K. Ueguchi-Tanaka M. Shimada Y. Nakazono M. Watanabe R.. (2006). GAMYB controls different sets of genes and is differentially regulated by microRNA in aleurone cells and anthers. Plant J. 47, 427–444. 10.1111/j.1365-313X.2006.02795.x 16792694

[B55] Voinnet O. (2009). Origin, biogenesis, and activity of plant microRNAs. Cell 136, 669–687. 10.1016/j.cell.2009.01.046 19239888

[B56] Wang F. D. Li L. B. Liu L. F. Li H. Y. Zhang Y. H. Yao Y. Y.. (2012). High-throughput sequencing discovery of conserved and novel microRNAs in Chinese cabbage (*Brassica rapa* L. ssp. pekinensis). Mol. Genet. Genomics 287, 555–563. 10.1007/s00438-012-0699-3 22643909

[B57] Wang J. W. Wang L. J. Mao Y. B. Cai W. J. Xue H. W. Chen X. Y. (2005). Control of root cap formation by microRNA-targeted auxin response factors in Arabidopsis. Plant Cell 17, 2204–2216. 10.1105/tpc.105.033076 16006581 PMC1182483

[B58] Wang Y. Liu W. Shen H. Zhu X. W. Zhai L. L. Xu L. (2015). Identification of radish (*Raphanus sativus* L.) miRNAs and their target genes to explore miRNA-mediated regulatory networks in lead (Pb) stress responses by high-throughput sequencing and degradome analysis. Plant Mol. Biol. Rep. 33, 358–376. 10.1007/s11105-014-0752-y

[B59] Wang Z. H. Zou Y. J. Li X. Y. Zhang Q. Y. Chen L. Wu H.. (2006). Cytoplasmic male sterility of rice with boro II cytoplasm is caused by a cytotoxic peptide and is restored by two related PPR motif genes via distinct modes of mRNA silencing. Plant Cell 18, 676–687. 10.1105/tpc.105 16489123 PMC1383642

[B60] Wang Z. J. Huang J. Q. Huang Y. J. Li Z. Zheng B. S. (2012). Discovery and profiling of novel and conserved microRNAs during flower development in *Carya cathayensis* via deep sequencing. Planta 236, 613–621. 10.1007/s00425-012-1634-x 22481137

[B61] Wang Z. W. Zhang Y. J. Xiang C. P. Mei S. Y. Zhou Y. Chen G. P.. (2008). A new fertility restorer locus linked closely to the Rfo locus for cytoplasmic male sterility in radish. Theor. Appl. Genet. 117, 313–320. 10.1007/s00122-008-0776-5 18542910

[B62] Wei L. Q. Yan L. F. Wang T. (2011). Deep sequencing on genome-wide scale reveals the unique composition and expression patterns of microRNAs in developing pollen of *Oryza sativa*. Genome Biol. 12:R53. 10.1186/gb-2011-12-6-r53 21679406 PMC3218841

[B63] Wei M. M. Wei H. L. Wu M. Song M. Z. Zhang J. F. Yu J. W.. (2013). Comparative expression profiling of miRNA during anther development in genetic male sterile and wild type cotton. BMC Plant Biol. 13:66. 10.1186/1471-2229-13-66 23597285 PMC3639194

[B64] Xie C. Mao X. Z. Huang J. J. Ding Y. Wu J. M. Dong S.. (2011). KOBAS 2.0: a web server for annotation and identification of enriched pathways and diseases. Nucleic Acids Res. 39, W316–W322. 10.1093/nar/gkr483 21715386 PMC3125809

[B65] Xing S. P. Salinas M. Höhmann S. Berndtgen R. Huijser P. (2010). miR156-targeted and nontargeted SBP-box transcription factors act in concert to secure male fertility in Arabidopsis. Plant Cell 22, 3935–3950. 10.1105/tpc.110.079343 21177480 PMC3027167

[B66] Xu L. Wang Y. Xu Y. Y. Wang L. J. Zhai L. L. Zhu X. W.. (2013b). Identification and characterization of novel and conserved microRNAs in radish (*Raphanus sativus* L.) using high-throughput sequencing. Plant Sci. 201, 108–114. 10.1016/j.plantsci.2012.11.010 23352408

[B67] Xu L. Wang Y. Zhai L. L. Xu Y. Y. Wang L. J. Zhu X. W.. (2013a). Genome-wide identification and characterization of cadmium-responsive microRNAs and their target genes in radish (*Raphanus sativus* L.) roots. J. Exp. Bot. 64, 4271–4287. 10.1093/jxb/ert240 24014874 PMC3808317

[B68] Yamaguchi A. Wu M. F. Yang L. Wu G. Poethig R. S. Wagner D. (2009). The microRNA-regulated SBP-Box transcription factor SPL3 is a direct upstream activator of *LEAFY, FRUITFULL* and *APETALA1*. Dev. Cell 17, 268–278. 10.1016/j.devcel.2009.06.007 19686687 PMC2908246

[B69] Yan J. J. Zhang H. Y. Zheng Y. Z. Ding Y. (2015). Comparative expression profiling of miRNAs between the cytoplasmic male sterile line MeixiangA and its maintainer line MeixiangB during rice anther development. Planta 241, 109–123. 10.1007/s00425-014-2167-2 25228384

[B70] Yang J. H. Liu X. Y. Xu B. C. Zhao N. Yang X. D. Zhang M. F. (2013). Identification of miRNAs and their targets using high-throughput sequencing and degradome analysis in cytoplasmic male-sterile and its maintainer fertile lines of *Brassica juncea*. BMC Genomics 14:9. 10.1186/1471-2164-14-9 23324572 PMC3553062

[B71] Yasumoto K. Terachi T. Yamagishi H. (2009). A novel Rf gene controlling fertility restoration of Ogura male sterility by RNA processing of orf138 found in Japanese wild radish and its STS markers. Genome 52, 495–504. 10.1139/G09-026 19483769

[B72] Yu B. Yang Z. Y. Li J. J. Minakhina S. Yang M. C. Padgett R. W.. (2005). Methylation as a crucial step in plant microRNA biogenesis. Science 307, 932–935. 10.1126/science.1107130 15705854 PMC5137370

[B73] Yu R. G. Wang Y. Xu L. Zhu X. W. Zhang W. Wang R. H.. (2015). Transcriptome profiling of root microRNAs reveals novel insights into taproot thickening in radish (*Raphanus sativus* L.). BMC Plant Biol. 15:30. 10.1186/s12870-015-0427-3 25644462 PMC4341240

[B74] Zhai L. L. Xu L. Wang Y. Huang D. Q. Yu R. G. Limera C. (2014). Genome-wide identification of embryogenesis-associated microRNAs in radish (*Raphanus sativus* L.) by high-throughput sequencing. Plant Mol. Biol. Rep. 32, 900–915. 10.1007/s11105-014-0700-x

[B75] Zhang Z. B. Zhu J. Gao J. F. Wang C. Li H. Li H.. (2007). Transcription factor *AtMYB103* is required for anther development by regulating tapetum development, callose dissolution and exine formation in Arabidopsis. Plant J. 52, 528–538. 10.1111/j.1365-313X.2007.03254.x 17727613

[B76] Zuker M. (2003). Mfold web server for nucleic acid folding and hybridization prediction. Nucleic Acids Res. 31, 3406–3415. 10.1093/nar/gkg595 12824337 PMC169194

