# Supplementary figures and images for: Identification of microRNAs and Their Target Genes Explores miRNA-Mediated Regulatory Network of Cytoplasmic Male Sterility Occurrence during Anther Development in Radish (Raphanus sativus L.)
Source: Front Plant Sci. 2016 Jul 22;7:1054. doi: 10.3389/fpls.2016.01054 (PMC4956657; doi:10.3389/fpls.2016.01054)

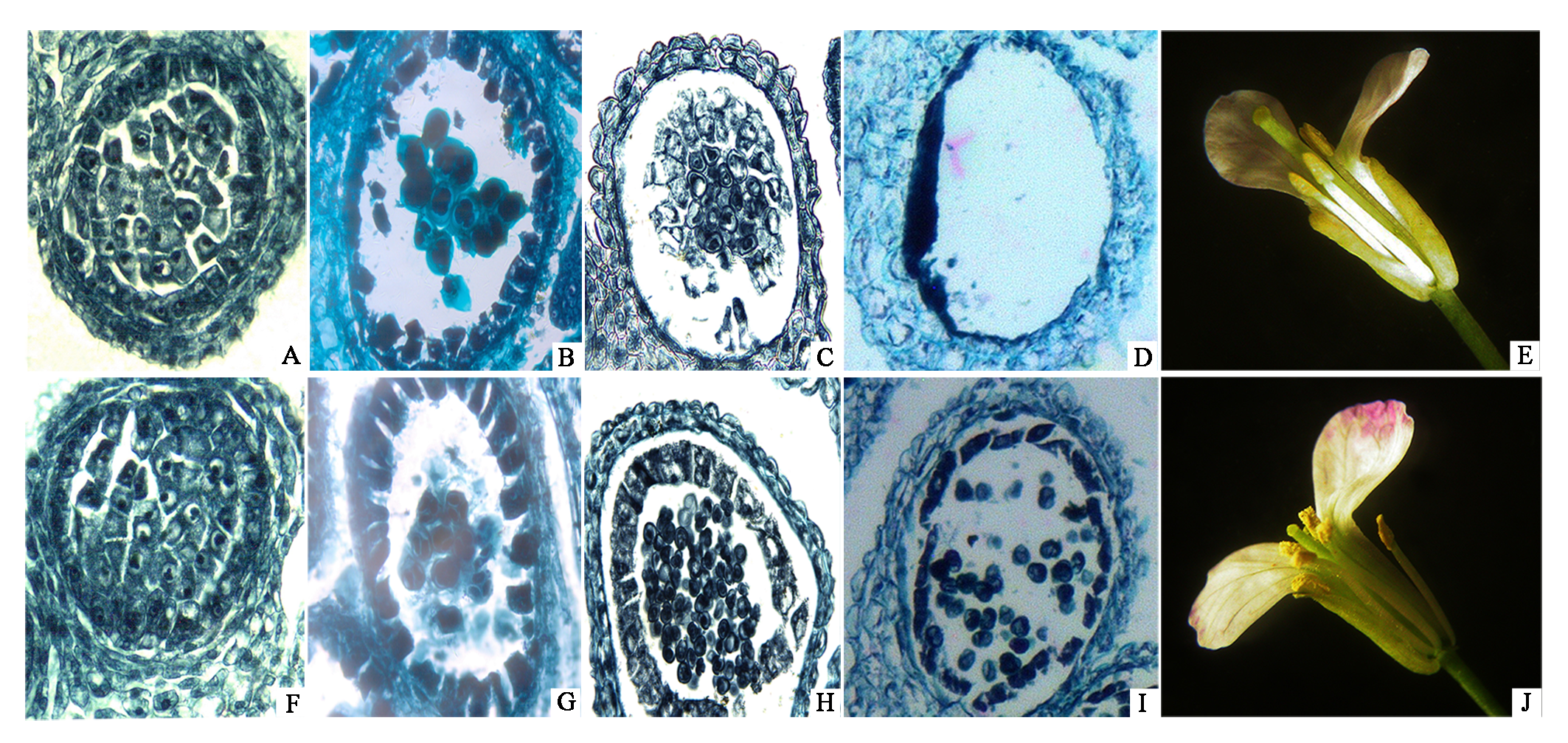

Supplement: Figure S1 — Micrographs of anthers at different developmental stages in the CMS line ‘WA’ (A–E) and its maintainer line ‘WB’ (F–J). Panels (A,F) Meiosis stage. Panels (B,G) Tetrad stage. Panels (C,H) Early microspore stage. Panels (D,I) Pollen stage. Panels (E,J) Flower morphology. [file Image1.TIF]

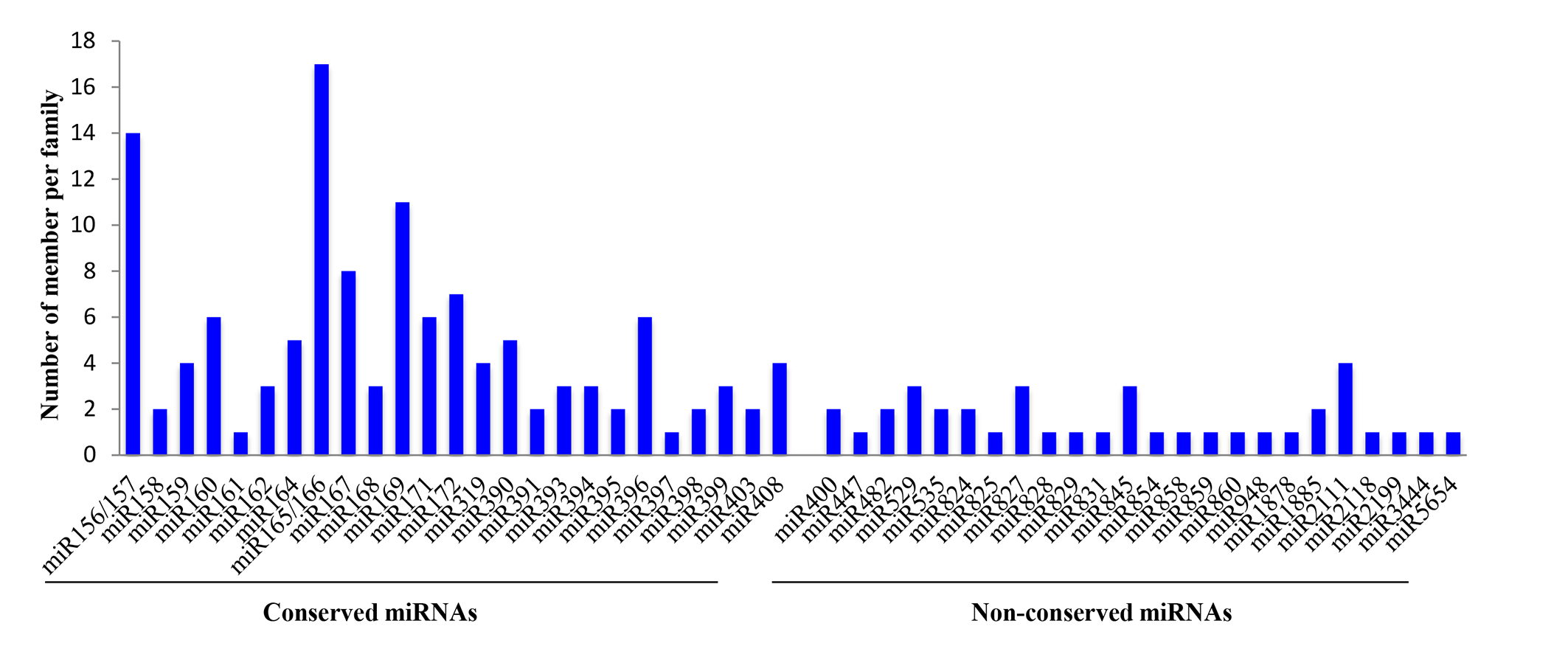

Supplement: Figure S2 — Distribution of known miRNA family members identified in radish. [file Image2.TIF]

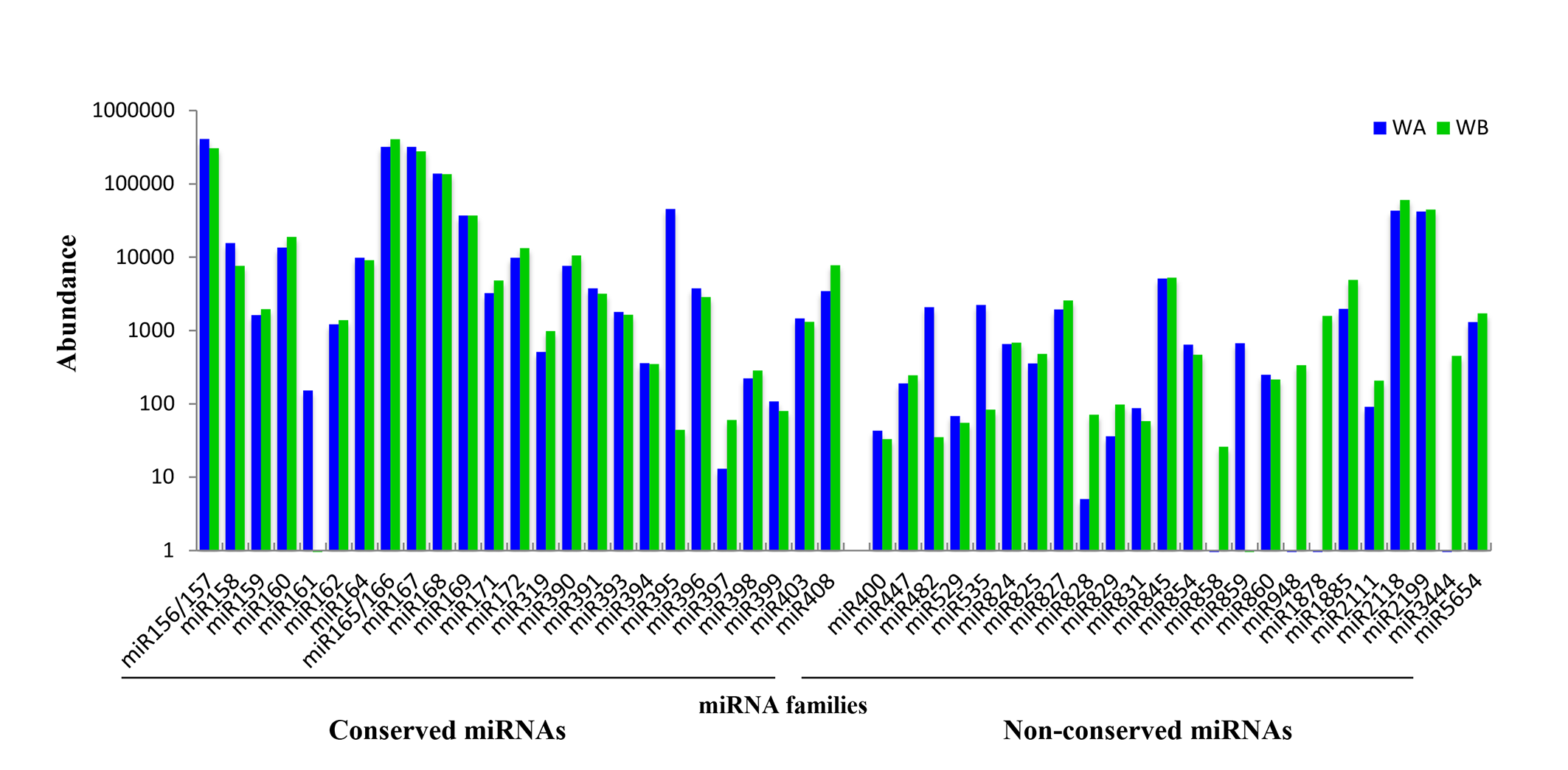

Supplement: Figure S3 — Abundance of each known miRNA family in radish. [file Image3.TIF]

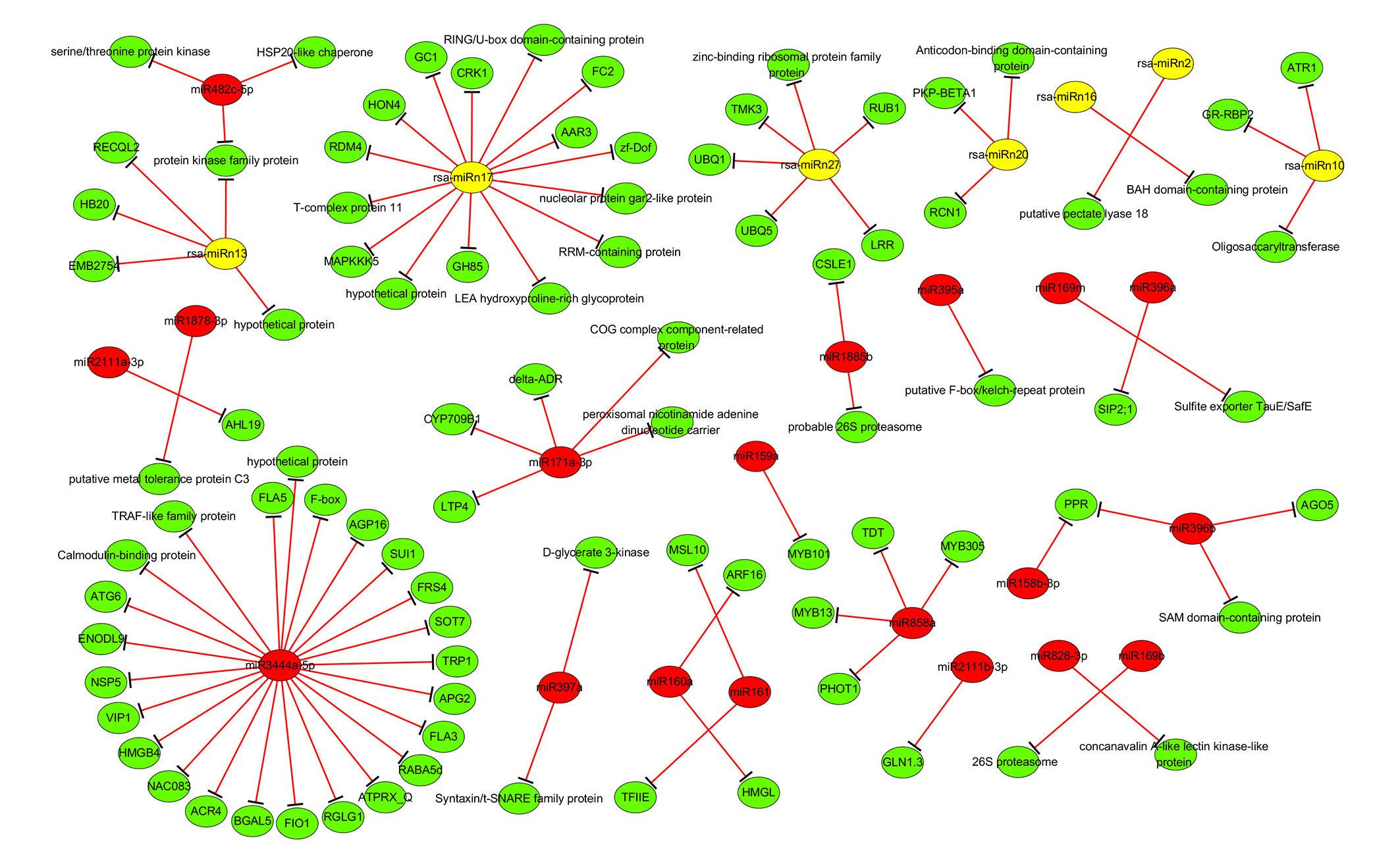

Supplement: Figure S5 — The miRNA mediated regulatory network constructed by Cytoscape_v3.2.1. The red, yellow and green ellipses represent the know miRNAs, potential novel miRNAs and target genes, respectively. [file Image5.tif]
