# Supplementary material for: Identification of microRNAs and Their Target Genes Explores miRNA-Mediated Regulatory Network of Cytoplasmic Male Sterility Occurrence during Anther Development in Radish (Raphanus sativus L.)
Source: Front Plant Sci. 2016 Jul 22;7:1054. doi: 10.3389/fpls.2016.01054 (PMC4956657; doi:10.3389/fpls.2016.01054)
Supplement: Figure S4 — Precursor sequences and the predicted second structures of novel miRNAs in radish. The mature miRNAs are in red and miRNA*s are in blue (“.” represent base mismatches, “(” represent base matches). [file Image4.PDF]

FIGURE S4 | Precursor sequences and the predicted second structures of novel miRNAs in radish. The mature miRNAs are in red and miRNA*s are in blue (“.” represent base mismatches, “(”represent base matches)

rsa-miRn1-precursor

GUCGCUCCCAAUACUCUCAAGAUUUUGCACAUGACAAACUAUCAAAAGAGACCUC
ACUCAUACAAAUCAAUGUAGGCUUCAAAAAAUGGUAAGGGUUUUAGGGUAAGGGA
GUGCCA

(.((((((((..((((((.(((.((((((.(((...............(((......))).((((......))))...........))))))))).))).)))))).))))))).).

**********AUACUCUCAAGAUUUUGCACAUG************************************
****

rsa-miRn2-precursor

GCUAACUCCACUACAAAAGUGUACUGCGGCAAUUCGUAUGUUAGCCUAUGGUUGU
GCGGGUGACAUGGUGGACGAAUAUCUCCGCAUGGGUGAGUCCACUGCGUUAUUGU
GUUUGGAAAAAUUUACUCAAGGAAUCAUUGAUUUAUAUGGAGAGAAGUAUCUACG
AAAACCCACACCUGAAGAUCUUCAACGACUACUUGAUAUCGGAGAGGUACGCGGU
UUCCCUGGGAUGAUAGGAAGCAUUGACUGUAUGCAUUGGGAGUGGAAAAAUU

......((((((.(...((((((.(((((((((.(...(((((.((((.((..(((.((((....(.(((((......((((((..((((((((((((..(((......))).))))....))))))))).
.(((((...)))))...))))))......))))).)..)))).)))(((.....((((..(((..........)))..))))....)))...)).)))).)))))....).)))).)))))))))))..))
)))))......

****
****
****
CAUUGACUGUAUGCAUUGGGAG*******

rsa-miRn3-precursor

GGCGGUCGUCGGUAUAUUCCGACGACAAUUCCGACGAAAGCCCGACAAAAAUUAU
AACCGUUACGGUCGUCGGAAUUCCGUCGGAAUAUACCGACGCCGU

.((((.((((((((((((((((((..((((((((((((....(((...................))))))))))))))).)))))))))))))))))))))

**************UAUUCCGACGACAAUUCCGACG**********************************
****

**UCGGAAU
UCCGUCGGAAUAUA**********

rsa-miRn4-precursor

UUGGUCAAGUGUAAUGUAUGUAGUCCAAUCUAUUGAAGUACUAGCACACCAUCUC
UAGUGAGGUGAUGUGGUUUGAGUAGUUCAAUACAUUGGACUACAUAUAUUACAUU
UGAUCAA

.(((((((((((((((((((((((((((.(((((((.(((((.((((.((((((....)))))).)))).)...)))).))))))).)))))))))))))))))))))))))))).

************AAUGUAUGUAGUCCAAUCUAU*************************************

****

****

*******ACAUUGGACUACAUAUAUUAC**********

rsa-miRn5-precursor

CUCAUCGUAGGAGAAAACGCUUCCAUAUCUAGCAGUAGGUUAUUGGUUAUUGGUG
UCACCCUUGACCAGAUCAGUUGGUGACAUUUUAUUAUGUGGGAGAGUUGUUAAAA
GGUGAGAUAUUUGAUUCGAGGGAUGCCAAGCACACCAAUAACCUACCGAUAGAUG
UGGAAGCGUUUCCGGUGAA

.(((((...(((...((((((((((((((((.(.((((((((((((((..(((((((..(((((((.(((((((.((..(((((((((.........)))).))))))..)).)))....)))))..))
))))))))))))....))))))))))))).).)))))))))))))))))))))))).

******************GCUUCCAUAUCUAGCAGUAGG*******************************

****

****

****

**UAC
CGAUAGAUGUGGAAGCGU**********

rsa-miRn6-precursor

AUGGCCGUUUAGGCCAAGGAAGUUUGAGGCAAUAACAGGUCUGUGAUGCCCUUAG
AUGUUCUGGGCCGCACGCGCGCUAC

.(((((((...((((.((((.((((((((((...(((....)))..)).)))))))).)))).))))....))).))))).

**********AGGCCAAGGAAGUUUGAGGCA***************************************

****

rsa-miRn7-precursor

CCCAUGAUGAUUUGCGUGAGUAUGUGGAUGUGUAUCUGCAUGUCCGCCACAUCUG
UUACUCAUGAAAAUCAUCAUACACACGAAAAUCACAUCCACAUACUCACGAAAAU
CAUAUGGGA

((((((.((((((.((((((((((((((((((.....((......))....(((((.....(((.....))).....))).))....)))))))))))))))))).)))))))))))).

**********UUUGCGUGAGUAUGUGGAUGU**************************************

****

****

***********AUCCACAUACUCACGAAAAUC********

rsa-miRn8a-precursor

AUAAUAAGAGUAAACAGAAUCACCUUGUCUGUCCAAAACAAGAAGAUAAAUAUAC

ACAUUCGUGUACUGAAAUUACAUAUGGUACGAUUGCUUAGCACUGGAAGCCAGGA

GGAACUUGUUUGCUGCAAACAU

........(((((((((..((..((((.((.((((.......(((...(((.(((.(((..(((((.......)))))))))))..))).))).....)))))).)))).))..)))))))))........

****

**********************AAGCCAGGAGGAACUUGUUUG**********

rsa-miRn8b-precursor

AUAAUAAGAGUAAAUAGAAUCACCUUGUCUGUCCAAAACAAGAAGAUAAAUAUAC

ACAUUCGUGUACUGAAAUUACAUAUGAUACGAUUGCUUAGCACUGGAAGCCAGGA

GGAACUUGUUUGCUGCAAACAU

........(((((((((..((..((((.((.((((.................(((((....)))))......................(((...))).)))))).)))).))..)))))))))........

****

**********************AAGCCAGGAGGAACUUGUUUG**********

rsa-miRn9a-precursor

AGAGCUCUUCCCAUCCCGUUCAGUUCUCCUUUUGCUUCACAUUGGAUCAGUCUCGU

CAAGACUGGUUCGAUACGAAGCAAACGAGAAUUGAACGGAACAAAGCACA

...(((.........(((((((((((((..((((((((..(((((((((((((.....)))))))))))))..)))))))).))))))))))))).....)))...

****************CGUUCAGUUCUCCUUUUGCUUC********************************

****

***AG

CAAACGAGAAUUGAACGGA**********

rsa-miRn9b-precursor

GGAGCUGGAGAGUUCUUCCCAUCCCGUUCAGUUCUCCUUUUGCUUCACAUUGGAU

CAGUCUCGUCAAGACUGGUUCGAUACGAAGCAAACGAGAAUUGAACGGAACAAAG

CACA

...(((((((....)))).....(((((((((((((..((((((((..(((((((((((((.....)))))))))))))..)))))))).))))))))))))).....)))...

************************CGUUCAGUUCUCCUUUUGCUUC***********************

****

****

****AGCAAACGAGAAUUGAACGGA**********

rsa-miRn10-precursor

AAACAAGUCGGCGUCCCCGACAUGGUCGUCUUCCUCAACAAGGAGGAUCAGGUCG

ACGACGCCGAGCUGCUC

...((..((((((((..((((.(((((.((((........)))).))))).))))..))))))))..))...

**********GCGUCCCCGACAUGGUCGUCU***************************************
**

rsa-miRn11-precursor

GCUACUGAAGCAGAGAGGCUAUCUCUUCUCUCCCGCUUGAAAACUGUCCCUGCUGU
GAACGCCUUUGGGAACGUUGAUGUGCUGGGGAAAGUUGAUCGUCUCUCUGACAAU
GG
.(((.((...(((((((((.(((.(((.(((((.((.....(((..((((....((....))....))))..))).....)).))))))))..))).)))))))))).)).)))
****
UGGGGAAAGUUGAUCGUCUCU*******

rsa-miRn12-precursor

UUAACUGGAUCCCAUGUGUGGUCGUUGCUUUUUUUUUGGACAGAAAGAACAUCAA
AAGAUUUUUUUUUUAAACAGUGGAUUUGAUAUUCAUUAGGGAAUUUCAUCGAACA
CGUUAACGACAAUUCUAAAAGAAGACUACAACACUCGAAAACAGAUCU
......(((((...(((.((((.((((.(((((((((((((.((((((((.(((....))).))))))))).....(((....(((.((((......)))).))).....)))...........))))))))
))))...)))))).))...))))))))
****
**AAAGAAGACUACAACACUCGA*
****

rsa-miRn13-precursor

CUUUUGGGCCGGUUGUGGGCGAGGAGGGAUUCGAACCCCCGACACCGUGGUUCGU
AGCCACGUGCUCUAAUCCUCUGAGCUACAAGCCCCACCCCGUCUCCACUGGAUCUG
UUCCCAGGAGUACCCUACAAAAAAAGGAACCUUUCCUCUCCCCAGCCAUUUCGGGU
UAAGAAGA
(((((..((((((((.(((.((((((((...((((((..((....)).))))))...((..((((((.........)))).))............((((((...(((.....)))..)))).))..........
..))...)))))))).))))))))......)))..))))).
**********GGUUGUGGGCGAGGAGGGAUU**************************************
****
****

rsa-miRn14-precursor

UGUGAAAAACGAGUGGAAACUUGUGACUCGGGUUGAGGAAGGACCCGAGGCUCGU
AGCUUCCACUCCAUGGCUUCG
..........((((((((.((((.(.((((((((.......)))))))).).)).)).))))))))..........
**********GAGUGGAAACUUGUGACUCGGGU************************************
****

rsa-miRn15-precursor

CAUGGACAAAGUCUCAUUUGAGGCGUCAGUAGAUUUUGUAUGUGCUGACUGUUCG
AAGAGGCCGCCUGUUCGAA

..((((((..(((((.((((((..(((((((.((.....)).)))))))..)))))))))))....))))))..

UGCUGACUGUUCGAAGAGGCCG***
*****

rsa-miRn16-precursor

UCGUCUGUAUCAGAGUUUCCUUUUCUCGUUCGCCGGGAACCGAUCUGACUGGUUA
GGGUUUCUCGUUCGCCUGGAACCGAUCUGACUGGUCAGGGUUUCUCGUUCGCCUG
GAACCGAUCUGACUGGUUAGGGUUUCUCGUUCGCCUGGAACCGAUCUGACUGGUU
ACAUCGAGUUUUAUAGCAGAAACAGAGAGUAAGGCUUCAAGAAGAAGAGGCUCUG
AACAAGAUUC

..((((...((((((((((.((((((.(...(((...((((...(((((..((((((((((..(....)..))))))...)))))..)))))))))((((.(((((.((((((((((.((((..((
((((((((((..(....)..))))))...)))))..)))).)))).))))))..)).)))...))))....)))..).)))))).))))))))))...))))..

****
****
CUUCAAGAAGAAGAGGCUCUG*****
**

rsa-miRn17-precursor

AGCCGGAGUGGCGGUUGCGGUUGCGGUUGCGUUUUCUCACCCAUCCAUCCGAAGC
AGCCUCACUAUGUAAUCUUGAAAACUGCACAGAUCUCUGUGAAUCGUGUGGCAAC
AACACUCUUGGAUGACGAGGACGAAACGUUGAGUAGGAACAGGAAGAGGAAGCAG
AUGCACUCAUGGCUGGGCAAUUCAUCGCAAUUGAACUUGGGACA

.(((.((((..((((((((((.(.((((((......(((.((((.((((....((..((((....(((..(((((..(((..(((((....)))))....(((((....).)))).((((.....))))..
......)))...))))))))....))))..)).)))).....)))).)))))))))))))))))))).)))).)).).

**********GCGGUUGCGGUUGCGGUUGC**
****
****

rsa-miRn18-precursor

CAGAAUCAACAGACUUGGAGCGCUCGAUGGCCGCAUUUUCCAGCUUCAAAAUGGU
GUCAUCGUUGCGCUGGACCAGCUCAUUUGCUCGCAUCUCACGCCUUUUCUCUCUUA
GUUGCCUUCUGU

(((((.((((.((.(((((((((.((((((((.((((((........))))))).))))))))..)))))...)))).))........((.......))............))))..))))).

**********AGACUUGGAGCGCUCGAUGGC***************************************
****

rsa-miRn19-precursor

CUGUGAGUUUCAGAACGAUAUAAAAGAUCAUGGAUACAGUAAAGAGAUUUAACUC
AAAUGAGGCUAGUAUAUCUAUGGCCUUUAUAUCGUAUUCGAAAUUGACAA

.(((.((((((..(((((((((((.(..((((((((...((((....)))).(((....))).......))))))))..))))))))))).)..)))))).))).

************GAACGAUAUAAAAGAUCAUGGA***********************************
****

***UAUG
GCCUUUAUAUCGUAUUCG**********

rsa-miRn20-precursor

AUUGUCGAUCAAUCGGUUGUAGAGAAGACGAUUCGUCCAGGGCUUGUGGAGCUAA
GCGAGGAUCCAGAUGUUGAUGUCAGGUUUUUUGCAAACCAAGCUCUUCAGUCUAU
UGAUAACGUGAUGAUGUCCAGCUAAAAAAACAAAUUUUAAAUCCUUGUCUAUCUC
UCUCUCCCUGGUUUUCUAUUUGAUGCAAUAUUAUGAACUUUCAAGACUUUGUAAC
GUAAGUAAGUGUGAACUCUUGUGUUCUGUUUUUCAUUUCAUGCACUCGGAAUUUG
UCUACUGUCUUGCAACUAGUGUGGUUCUUUUU

......(((((((..(((((((((.((((((.....((((((...(.((((.((..(((((((..(((..(((......(((((.....))))).((((.....(((.(((........))))))....)))
).......)))..)))..))))))))..)).))))))...)))))))........(((((((((.......((((...(((((.((..((.(........)))..)).))))).)))).(.....)......)))).)
))))..)))))).))...))))))))..)).))))))......

**********AAUCGGUUGUAGAGAAGACGAU*************************************
****
****
****
*

rsa-miRn21a-precursor

UUGGAGAAGACAGAGACAGAGAGGAGAAAGGAAUCGUGUAAGCAAGCACAAAACU
UUCCUUUCUUCUAUCUCUAUCUCUCUCCUUUCGACCAGA

((((.(((((.(((((.((((((((((((((((..((((......))))......))))))))))))..))))).))))).))..)))..)))).

**********CAGAGACAGAGAGGAGAAAGGAA************************************
****

rsa-miRn21b-precursor

UUGGAGAAGACAGAGACAGAGAGGAGAAAGGAAUCGUGUAAGCAAGCACAAAACU
UUCCUUUCUUCUAUCUCUAUCUCUCUCCUUUCGAU

...((((.((.(((((.((((((((((((((((..((((......))))......))))))))))))..))))).))))).)).))))...

**********CAGAGACAGAGAGGAGAAAGGAA************************************
****

rsa-miRn22-precursor

CGACUUUGCUUGCUUCUUUCUCAGCCGUUUCAAGUAUCGGGCUGGUGGGUUUCAG
AGGUUCUGGUUGCUGCUCUUUGGGUAUGCUUUCAUAUUCCUCUCUUCCUUUUCGG
CUUUAAGUUCUUCAAUGUCAAUGGAGGUGAAAAACUCGGGGAAAUCAUCACGAAC
UAGCUACUUAGGUGAUGGCAUGGGAUGUUUCGGUAACCGAAGAAAUGAGGUGAAC
GCCACCUUCCGUGGUUACCAUCAACGGUAGAGAACGGGGGUUGAUAGUCU
.((((...(..((((((((((((.((((((..(((((..(((((((.(((....(((((...(((..((((((.....)))).))..)))....)))))...)))..)))))))..(((((....(((...
.(.(((........))).)......)))...)))))...))))).((((((..(((((....((((((...))))))....((((((.....))))))))))))))))...)))))).))))).)))))
).)..)))).
****
****
****
AUCAACGGUAGAGAACGGGGG********

rsa-miRn23-precursor

UUUUUUGUAUAUAUCUCUCUCUCUCUCUCGCUUUCAAUCACGAUCUACGAUGA
UCUUACUGGUGCUGCAUAACGUAAAACGUAAAAUGCGAGAGGAUCGAAGCUACGG
AAGCUCAAGGGCGAAGGAUAGAGAGAGAGAGAGAGAUAAUUCAAAACU
..(((((....((((((((((((((((((..(((((.....((((((.(....(((.....)))...((((.(((.....)))...))))..).)))))).((((.....)))).......)))))..))
)))))))))))))))))...)))))..
****
**AUAGAGAGAGAGAGAGAGAUA
****

rsa-miRn24-precursor

AGAUUCGCUUGGUGCAGUUCGGGACUGAUUGGCUGACACAACCACAUAGGUACUU
UUGCCUUAUUGGUUUGUAAGCAUGGAUUGGCUCCCGCCUUGCAUCAACUGAAUCG
.((((((.(((((((((..((((((..(((.(((.(((.(((((...(((((....)))))...)))))))).)))...)))..).)))))..))))))))).)))))).
**********GGUGCAGUUCGGGACUGAUUG***************************************
****
****
*AUUGGCUCCCGCCUUGCAUCAA********

rsa-miRn25-precursor

ACUGGAUGGAAUUUCAGUGAAUUAGACUGAGAAGAAUCUUGAAGUCCUAGCUUUU
UGUUCGAUACAAAAAAGUAAAGUAUGUAGGUCUAAAAUUUUUAGCCUGUUCUCCU
UUGGUAGUUUGACCGCGAAAUUUUUUUUCUGCA
...(((.((((((((.(((..((((((((.((((...............(((((((((.....)).))))))))........((((.((((.....))))))))).....))))..)))))))).))))))))
)))...)))...

****
*********************************GGUAGUUUGACCGCGAAAUUU**********

rsa-miRn26-precursor

UACCAACAUCACUUUCUGGGUGUUGCUGAAGGCUCUUGCUGAUUCUGGGAAGCUA
AAUCUGGCUCUCAACUACUUGGAGCGAAUGGAGGGGGAGUUUAGUGUGAAGCCAG
AGUCCACUCACCAUGGGGUAGUGAUCGAUGGUUUUGCCAGAACCAAAGACGCAAA
ACCUGGCCUUCGCUGUUGAGAGGGUGAAGCAACUUGUCCCUUUCCAGGGUCAGGA
AAUGUCAUUCAAUGCCAUACUGAGCAGGUGCUACCCACCCAGAUAUCCUACCAAAG
AAGAUCUGAAGUGGUUGAAA

.........(((((.((.(((((.(((((((((..((.((((((((((((((.........(((.(((.((.((..(((((((.((..(((..((((.(.(..((((((..(..((((.(((....))))
)))..)..))))))..)).)))).............)))..))))))))).)..)).)).))))))........)))))))))))))).))..))).))))..)).))))).)).)))))....((((.((
(((...((.....))...)))))..))))......

**********ACUUUCUGGGUGUUGCUGAAGGC************************************
****
****
****

rsa-miRn27-precursor

AGCGUCGACUCCUUCUGGAUGUUGUAGUCGGCGAGGGUGCGGCCGUCUUCCAGCU
GCUUCCCGGCGAAGAUCAGUCGCUGCUGGUCCGGAGGGAUUCCUUCCUUGUCCUGG
AUCUUGGCCUUGACGUUG

((((((((.(((((((((((...((((.((((..(((.((((((.(.....).))))))..)))...(.....).))))))))..)))))))))))..((.(((.......)))....))..)))))))).

**********CCUUCUGGAUGUUGUAGUCGG**
****
